# Adhesive organ regeneration in *Macrostomum lignano*

**DOI:** 10.1186/s12861-016-0121-1

**Published:** 2016-06-02

**Authors:** Birgit Lengerer, Elise Hennebert, Patrick Flammang, Willi Salvenmoser, Peter Ladurner

**Affiliations:** Institute of Zoology and Center of Molecular Bioscience Innsbruck, University of Innsbruck, Technikerstr. 25, A-6020 Innsbruck, Austria; Biology of Marine Organisms and Biomimetics, Research Institute for Biosciences, University of Mons, 23 Place du Parc, 7000 Mons, Belgium; Laboratory of Cell Biology, Research Institute for Biosciences, University of Mons, 23 Place du Parc, 7000 Mons, Belgium

**Keywords:** Lectin, Flatworm, Duo-gland system, Marine adhesion, TEM, Superresolution microscopy

## Abstract

**Background:**

Flatworms possess pluripotent stem cells that can give rise to all cell types, which allows them to restore lost body parts after injury or amputation. This makes flatworms excellent model systems for studying regeneration. In this study, we present the adhesive organs of a marine flatworm as a simple model system for organ regeneration. *Macrostomum lignano* has approximately 130 adhesive organs at the ventral side of its tail plate. One adhesive organ consists of three interacting cells: one adhesive gland cell, one releasing gland cell, and one modified epidermal cell, called an anchor cell. However, no specific markers for these cell types were available to study the regeneration of adhesive organs.

**Results:**

We tested 15 commercially available lectins for their ability to label adhesive organs and found one lectin (peanut agglutinin) to be specific to adhesive gland cells. We visualized the morphology of regenerating adhesive organs using lectin- and antibody staining as well as transmission electron microscopy. Our findings indicate that the two gland cells differentiate earlier than the connected anchor cells. Using EdU/lectin staining of partially amputated adhesive organs, we showed that their regeneration can proceed in two ways. First, adhesive gland cell bodies are able to survive partial amputation and reconnect with newly formed anchor cells. Second, adhesive gland cell bodies are cleared away, and the entire adhesive organ is build anew.

**Conclusion:**

Our results provide the first insights into adhesive organ regeneration and describe ten new markers for differentiated cells and tissues in *M. lignano*. The position of adhesive organ cells within the blastema and their chronological differentiation have been shown for the first time. *M. lignano* can regenerate adhesive organs *de novo* but also replace individual anchor cells in an injured organ. Our findings contribute to a better understanding of organogenesis in flatworms and enable further molecular investigations of cell-fate decisions during regeneration.

**Electronic supplementary material:**

The online version of this article (doi:10.1186/s12861-016-0121-1) contains supplementary material, which is available to authorized users.

## Background

Flatworms (Platyhelminthes) possess the extraordinary capacity of regeneration. Recently, the molecular foundation of the stem-cell-based regeneration process has been intensely studied in planarians [1–4]. Several studies have addressed regeneration and stem cell regulation for the basal, free-living flatworm *Macrostomum lignano* [5–8]. *M. lignano* is able to regenerate its anterior-most region and any tissue posterior to the pharynx [5, 6]. After amputation, regeneration of the tail plate completes within 6 to 10 days [9]. In previous studies, the number of differentiated adhesive organs has been used as a marker for complete tail-plate regeneration [6, 9]. *M. lignano* is a small marine flatworm that was first described in 2005 [10]. The animal possesses approximately 130 adhesive organs in a half-moon shaped arc at the ventral side of its tail plate [9, 10]. Each organ consists of three cell types [11]: an adhesive gland cell, which secretes the glue to adhere animals to any substrate, and a releasing gland cell, which expels a releasing factor for detachment, both gland cells secreting their vesicles through a modified epidermal cell (the anchor cell). We use the term “adhesive organ” to refer to a cluster of one adhesive gland cell, one releasing gland cell, and one anchor cell, as defined by Tyler [12]. The simplicity of the system—i.e. three interacting cells, a fast regeneration time, and restricted localization in the tail—makes adhesive organs an optimal system for analysing regeneration. After tail-amputation, it is obvious that all adhesive organs have to be completely rebuilt from stem cells. This process requires the coordinated spatial and temporal differentiation of the three cell types. Furthermore, the outgrowing necks of one adhesive gland cell and one releasing gland cell must pair and together penetrate a newly forming anchor cell [11]. This has to occur independently about 130 times. Finally, the anchor cells must position themselves in a horseshoe-shaped arc at the ventral side of the tail plate. Such a developmental mechanism raises the question of whether *M. lignano,* and perhaps flatworms in general, have a defined developmental program for adhesive organ formation. This hypothesis leads to the conclusion that direct cellular interaction and an encompassing regulatory program are required for the formation of a functional adhesive organ. Alternatively, flatworms may show developmental plasticity with respect to adhesive organ formation. Thereby, flatworms must be able to integrate a newly differentiating stem cell into a partially injured organ. One problem in addressing this question is the absence of cell type-specific markers. Apart from some tissue- and cell type-specific antibodies for *M. lignano* [7, 13, 14], adhesive cell type-specific labelling is missing. In studies of several invertebrate species, lectins have been used as a marker for specific tissues [15–17]. Lectins are proteins with a high binding specificity to the oligosaccharide moieties found in glycoproteins, and they are widely used in biomedical research [18]. Moreover, lectins were successfully applied in the planarian flatworm *Schmidtea mediterranea* [17] and the sea star *Asterias rubens* [19] to label secretory adhesive cells. Therefore, we tested commercially available lectins for their ability to label *M. lignano* secretory cells.

Here, we present ten new markers for differentiated *M. lignano* cell types and tissues, nine lectins, and one cell-type specific antibody. We describe the morphology of regenerating adhesive organs using two of these markers (one lectin and the antibody), as well as with EdU staining and transmission electron microscopy. We show that adhesive gland and releasing gland cells differentiate earlier than their connected anchor cell. Before the anchor cell migrates to the epidermal surface and forms microvilli, it surrounds the necks of two fully differentiated gland cells. Partial amputation of anchor cells revealed that some adhesive gland cell bodies survive this injury and reconnect with a newly formed anchor cell. Our findings pave the way for further molecular analyses of cell-fate decisions during adhesive organ regeneration.

## Results

### Lectins as markers for differentiated cells and tissues of *Macrostomum lignano*

**Fig. 1:**
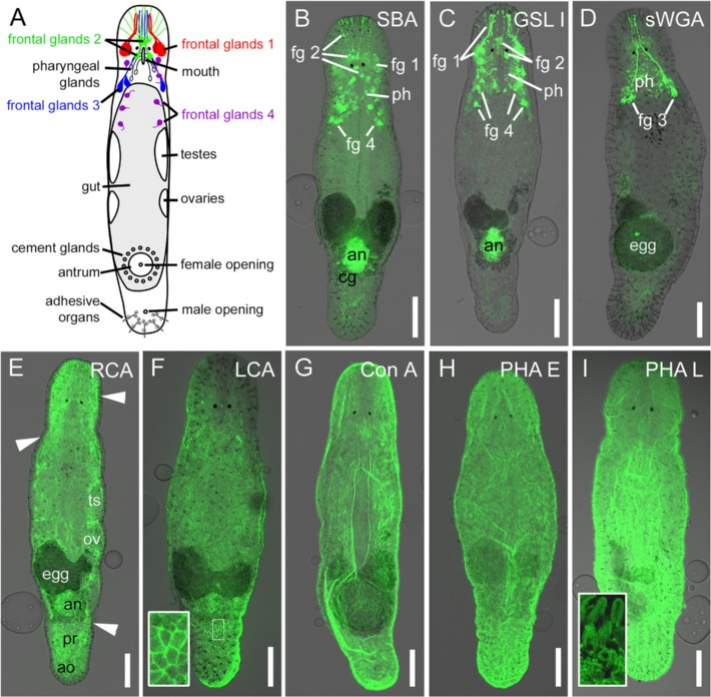
Schematic drawing and lectin labelling of *Macrostomum lignano.* **a** Schematic drawing of an adult specimen. **b-i** Confocal projections of lectin labelling imposed on DIC images. **b** SBA and (**c**) GSL I staining of frontal glands 1, 2, and 4, pharyngeal glands, and the antrum. **b** SBA additionally stains the cement glands. **d** sWGA staining of frontal glands 3 and pharyngeal glands. **e** RCA labelling. Arrowheads indicate the unstained epidermal layer. **f** LCA staining of epidermal junctions. Inset shows the epidermis at a higher magnification. **g** Ubiquitous Con A labelling of the epidermis. **h** PHA E and (**i**) PHA L staining of the glycocalyx of the epidermal microvilli. Inset illustrates stained microvilli of adhesive organs. *An* antrum, *ao* adhesive organs, *cg* cement glands, *egg* developing egg, *fg* frontal glands, *ov* ovaries, *ts* testes, *ph* pharyngeal glands. Scale bars: 100 μm

We tested 15 different biotinylated lectins (Table 1) for their ability to label the secretory gland cells of the adhesive organs in *Macrostomum lignano*. For a detailed overview of the oligosaccharide binding specificity of the selected lectins, see [19] (Additional file 1: Table S1). The negative control—skipping the lectin and using only the streptavidin-conjugate for labelling—led to a minimal general background. Out of the 15 tested lectins, 9 led to a labelling in distinct tissues (summarized in Table 1), including the epidermis, the female antrum, developing eggs, and various types of secretory glands. In the anterior part of the animal, four novel types of frontal glands were identified (Fig. 1a and Additional file 2: Figure S1). The large cell bodies of frontal gland type 1 were located lateral to the eyes. Their necks converged in the rostrum and discharged on the ventral side, slightly behind the anterior end (Additional file 2: Figure S1B). The cell bodies of frontal gland type 2 were positioned along the midline between the eyes. Their gland necks projected towards the anterior and lateral margin (Additional file 2: Figure S1C). Frontal gland type 3 necks formed a bundle in the middle of the rostrum. Their cell bodies were found posterolateral to the pharynx, in close proximity to rhammite glands [10] (Additional file 2: Figure S1D). All gland cell openings were located on the ventral side, while the cell bodies of frontal gland types 1, 2, and 3 were found dorsally. The exception was frontal gland type 4, which was positioned on the ventral side, with necks through the ventral epidermis. The cell bodies of frontal gland type 4 were located mediolaterally in the region between the level of the eyes and the tip of the testes (Additional file 2: Figure S1E).

**Table 1:** Lectin labelling of different cells and tissues in *Macrostomum lignano*

Lectin	Acronym	Number specimen	Overall	Epi-dermis	Adhesive glands	Frontal glands 1, 2, 4	Frontal glands 3	Pharyngeal glands	Testes	Developing eggs	Cement glands	Antrum	Prostate
*Lens culinaris* agglutinin	LCA	*n* = 25		+++^a^									
*Phaseolus vulgaris* erythro agglutinin	PHA-E	*n* = 28		+++^b^									
*Phaseolus vulgaris* leuco agglutinin	PHA-L	*n* = 27		+++^b^									
Succinylated wheat germ agglutinin	sWGA	*n* = 17					+++	++				++	
*Griffonia* (Bandeiraea) *simplicifolia* lectin I	GSL I	*n* = 23				+++		++		+++		+++	
Soybean agglutinin	SBA	*n* = 45				+++		++		+++	+	+++	++
Peanut agglutinin	PNA	*n* = 48			+++	+++		+++	+	++	++	+++	
*Ricinus communis* agglutinin	RCA	*n* = 24	++		+++	++				+++		+++	+++
Concanavaline A	Con A	*n* = 32		+++									
Wheat germ agglutinin	WGA	*n* = 23	~										
*Sambucus nigra* agglutinin	SNA	*n* = 16	~										
*Maackia amurensis* lectin II	MAL II	*n* = 18	~										
*Dolichos bilforus* agglutinin	DBA	*n* = 21	~										
*Sophora Japonica* agglutinin	SJA	*n* = 20	~										
*Ulex europaeus* agglutinin 1	UEA 1	*n* = 12	~										
neg. control (without lectin)		*n* = 36	~										

+weak labelling,++ intermediate labelling, +++ strong labelling, ~ unspecific background

^a^probably epidermal cell junctions

^b^glycocalyx of epidermal microvilli and modified microvilli of anchor cells

Soybean agglutinin (SBA) labelled frontal glands 1, 2, and 4, as well as the pharyngeal glands, the female antrum, the cement glands, and developing eggs in the antrum (Fig. 1b and Additional file 3: Figure S2). In most of the specimen (37 out of 45), SBA additionally labelled the prostate glands (Additional file 3: Figure S2). *Griffonia simplicifolia* lectin I (GSL I) led to a staining in the pharyngeal glands, the female antrum, and frontal glands 1, 2, and 4 (Fig. 1c and Additional file 4: Figure S3). In contrast to SBA, no staining was observed in the cement glands or prostate glands. Succinylated wheat germ agglutinin (sWGA) labelled the pharyngeal glands and frontal glands type 3 (Fig. 1d and Additional file 5: Figure S4). The necks of frontal gland type 3 proceeded in parallel with rhammites through the neuropil and the rostrum (Additional file 5: Figure S4B-C). *Ricinus communis* agglutinin (RCA) resulted in a ubiquitous staining of the whole animal, excluding the epidermis (Fig. 1e and Additional file 6: Figure S5). Some tissues, such as the female antrum, developing eggs, the prostate glands, and the secretory gland cells of the adhesive organs, appeared to be more strongly labelled than the rest of the animal (Fig. 1e and Additional file 6: Figure S5). Out of 15 lectins, four labelled the epidermal layer of the animals. *Lens culinaris* agglutinin (LCA) stained the outline of the epidermal cells, probably representing the epidermal cell junctions (Fig. 1f and Additional file 7: Figure S6A1-B3). Concanavalin A (Con A) led to an overall staining of the epidermal layer (Fig. 1g and Additional file 7: Figure S6C1-D3). *Phaseolus vulgaris* erythro (PHA-E) and leuco (PHA-L) agglutinins led to the same speckled staining of the epidermis (Fig. 1h-i). At higher magnification, hair-like structures on the epidermal surface became obvious, most likely representing the glycocalyx of epidermal microvilli (Additional file 8: Figure S7A-D). The rhabdite gland openings, which penetrate the epidermis, remained unstained (Additional file 8: Figure S7B). At the ventral side of the tail plate, a clear staining of the specialized microvilli of the adhesive organs was visible (Fig. 1i inset and Additional file 8: Figure S7D), representing their glycocalyx. The glycocalyx covers the epidermal surface, including microvilli and adhesive organs (Additional file 8: Figure S7C). Out of the tested lectins, the *Arachis hypogaea* peanut agglutinin (PNA) resulted in specific labelling of the adhesive gland cells in the tail plate, along with other tissues (described in the next section).

### Peanut agglutinin as an adhesive gland cell marker

**Fig. 2:**
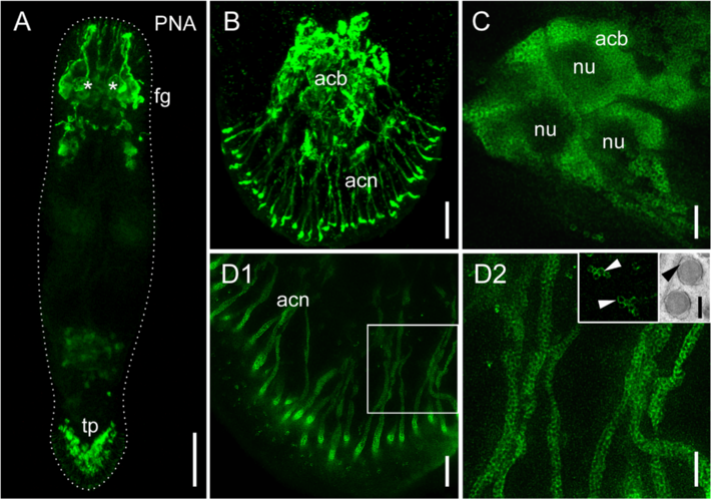
Fluorescence microscope-, confocal-, and gSTED images of PNA labelled *Macrostomum lignano*. **a** Overview of a PNA labelled adult animal. Asterisks indicate position of eyes. **b** Tail plate of a hatchling (confocal projection) with labelled adhesive gland cells. (**c**-**d**2) Projections of gSTED images. **c** Adhesive gland cell bodies with labelled vesicles in the cytoplasm, dark areas represent the nucleus. (**d**1) Gland cell necks filled with vesicles and (**d**2) detail thereof. Inset 1: detail of stained vesicles, arrowheads indicate the circular staining surrounding an unstained center. Inset 2: TEM image of adhesive gland vesicles, arrowhead indicates the lucid rim surrounding an electron-dense core. *Acb* adhesive gland cell bodies, *acn* adhesive gland cell necks, *fg* frontal glands, *nu* nucleus, *ph* pharyngeal glands, *tp* tail plate. Scale bars: (A) 100 μm, (B) 10 μm, (C,D2) 2 μm, (D1) 5 μm, (D inset) 200 nm

PNA stained the secretory glands in the tail, pharyngeal glands, the female antrum, developing eggs, and frontal secretory glands 1, 2, and 4 (Fig. 2 and Additional file 9: Figure S8). In about one third of the specimen (15 out of 48), the cement glands were also stained. Single individuals also showed a weak labelling of the centre of the testes (Fig. 2 and Additional file 9: Figure S8). In the tail plate, secretory gland cells with long necks were stained (Fig. 2a, b). Due to their location and appearance, they can be classified as the secretory gland cells of the adhesive organs [11]. To determine whether PNA labels the adhesive- and/or releasing gland cells of the respective organs, high resolution gated stimulated emission depletion (gSTED) microscopy was performed. To reduce specimen thickness, hatchlings were used for gSTED microscopy (Fig. 2c-d). The staining was restricted to vesicles measuring about 270 nm in diameter (Fig. 2d inset), a characteristic size for adhesive gland cell vesicles [11]. No vesicles of a smaller size or other stained structures were present in the tail plate. In almost all labelled cells, the cytoplasm of the cell bodies (Fig. 2c) and the gland necks (Fig. 2d1-2) were densely filled with vesicles, corroborating previous findings by transmission electron microscopy (TEM) [11]. Interestingly, the labelling of the vesicles appeared as a ring-like structure, leaving the centre of the vesicles unstained (Fig. 2d inset 1). In TEM images of cryo-processed specimen, the adhesive gland vesicles contained an electron-dense inner core and a more lucid outer rim surrounded by the vesicle membrane (Fig. 2d inset 2). According to these observations, PNA labelling was restricted to the lucid outer rim of the vesicles. PNA preferentially binds to galactosyl (β-1,3) N-acetylgalactosamine present in glycoconjugates (according to manufacturer’s information; Vector Laboratories). The labelling was drastically reduced when PNA was pre-incubated with its inhibitory monosaccharide D-galactose (Additional file 9: Figure S8D-E). These results indicate the presence of at least one glycoconjugate with a galactosyl (β-1,3) N-acetylgalactosamine residue in the adhesive gland cell vesicles. Due to its specific staining of the adhesive gland cell vesicles, PNA can be used as a marker for differentiated adhesive gland cells.

### An intermediate filament-specific antibody as an anchor cell marker

**Fig. 3:**
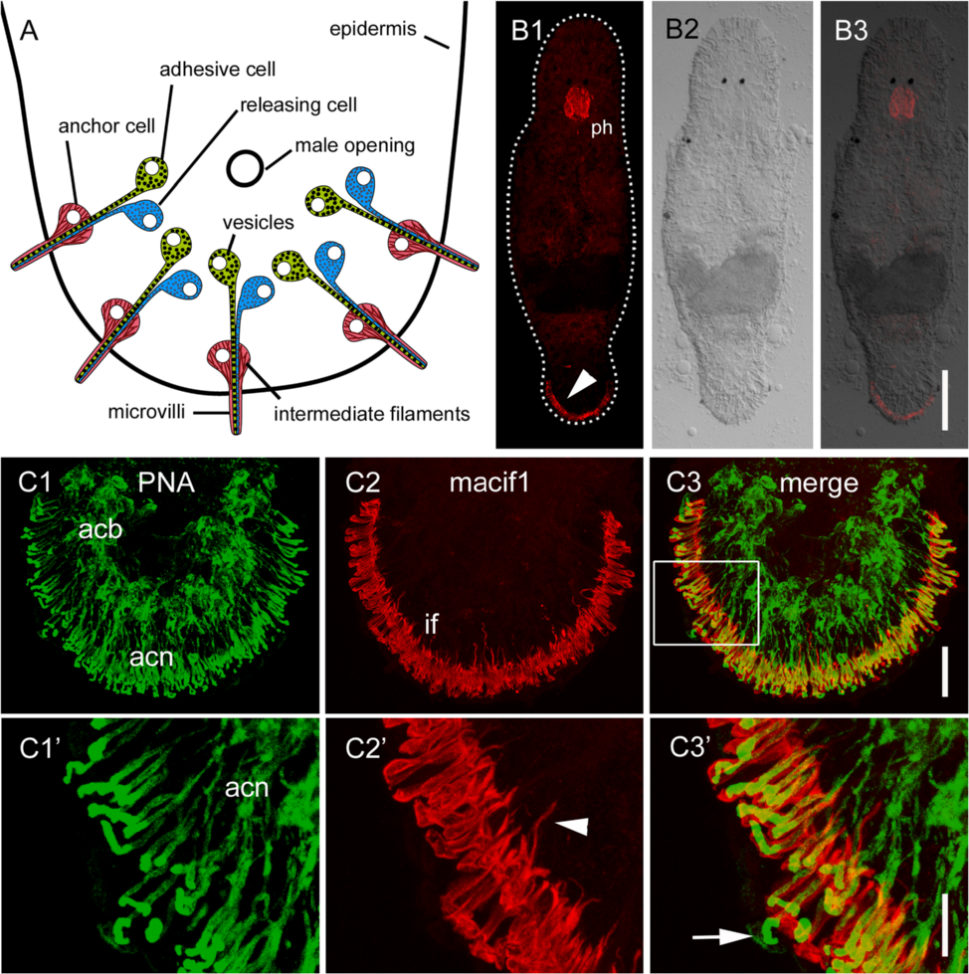
Schematic drawing and labelling of *Macrostomum lignano* adhesive organs. **a** Schematic drawing of adhesive organs at the tail plate. Note that the microvilli collar, protruding from the epidermis do not contain any intermediate filaments but actin filaments. (**b**1-3) Anti-Macif1 antibody staining of a whole specimen. Arrowhead indicates intermediate filaments in the anchor cells. (**c**1-3) Confocal projection of PNA- and antibody Macif1 double staining (boxed area in C’). (**c**1) PNA labelled adhesive gland cell bodies and -necks. (**c**2) Intermediate filaments in the anchor cells. Arrowhead indicates intermediate filament connections to the extracellular matrix. (**c**3) Overlay of PNA lectin and Macif1 staining. Note that the adhesive gland cell necks are surrounded by intermediate filaments of anchor cells. Arrow highlights adhesive gland cell necks at the area of microvilli collar. *Acb* adhesive gland cell bodies, *acn* adhesive gland cell necks, *if* intermediate filaments, *ph* pharynx. Scale bars: (B) 100 μm, (C1-3) 20 μm, (C1’-3’) 10 μm

Along with the adhesive gland cell, one adhesive organ consists of one releasing gland cell and one modified epidermal cell, called the anchor cell (Fig. 3a) [11, 12]. In a previous study, the intermediate filament variant Macif1 was found to be anchor cell specific and crucial for the cell’s functional integrity [11]. We have generated a polyclonal antibody specific for Macif1 (see Methods). The staining with this antibody resulted in the labelling of intermediate filaments in the anchor cells as well as in the pharynx (Fig. 3b). The structural organization of the adhesive organs was corroborated by PNA and Macif1 double staining (Fig. 3c). The adhesive gland cell bodies were located further anterior in the tail plate, and they formed long necks penetrating the anchor cells (Fig. 3c1). The cell bodies of the anchor cells were located below the body wall musculature and contained intermediate filaments (Fig. 3c2). Additionally, these filaments were also located in the cytoplasmic protrusions of the anchor cells, which extended to the extra cellular matrix (Fig. 3c2’) [11]. The modified microvilli of the anchor cells were strongly reinforced by actin filament bundles and could be stained with fluorescent phalloidin [11]. Accordingly, the external part of the adhesive organs were only stained by PNA and not by Macif1 (Fig. 3C3’). The Macif1 antibody in combination with PNA staining was then used for the morphological characterization of the anchor cells and adhesive gland cells during regeneration (see next sections). No marker for releasing gland cells is currently available.

### Adhesive organ regeneration after tail plate amputation

**Fig. 4:**
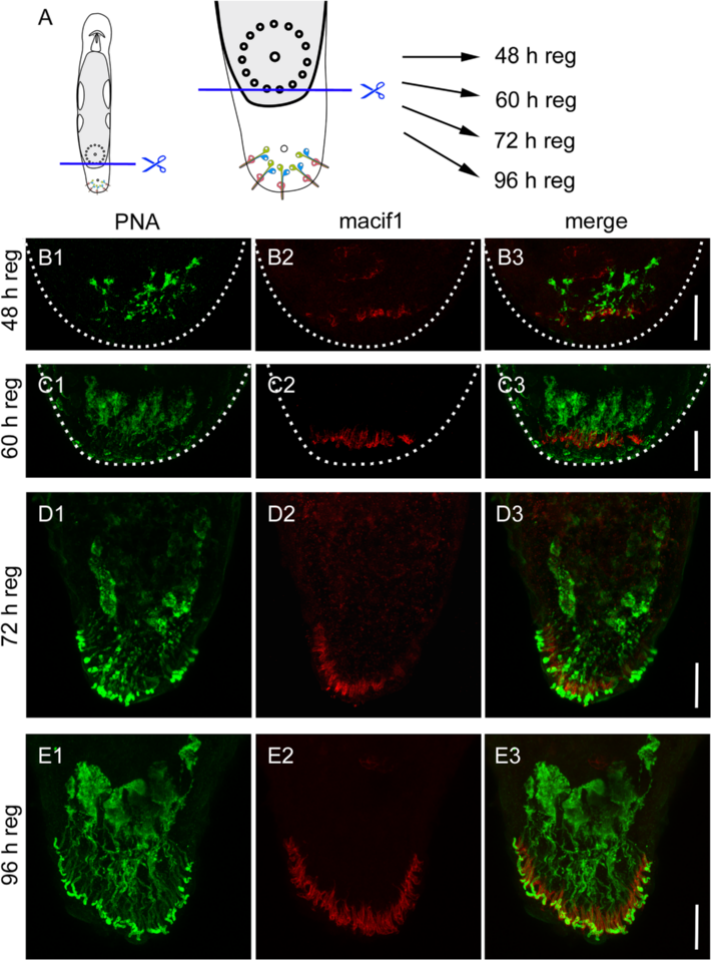
Regeneration of adhesive organs after tail plate amputation. **a** Schematic drawing of *Macrostomum lignano* with indication of the amputation level. **b**-**e** PNA lectin and Macif1 double staining of regenerating specimen after (**b**1-3) 48 h, (**c**1-3) 60 h, (**d**1-3) 72 h, and (**e**1-3) 96 h of tail plate regeneration. Images are Z projections of confocal stacks. Note that according to Egger et al. [9] the early regeneration blastema is slightly bend towards the ventral side. Therefore, in figures B1-C3 the location of the differentiated anchor cells appears to be within the tail plate. Dotted line indicates the outline of the blastema. Scale bars: 20 μm

Next, we aimed to elucidate how approximately 130 adhesive organs [9] are regenerated within one week. To remove the complete tail plate, including all adhesive organs, the animals were amputated posterior to the cement glands that surround the female genital opening (Fig. 4a). No differentiated adhesive gland cells or anchor cells were found until 36 h post amputation. From 48 to 60 h post amputation, cells labelled with PNA and Macif1 were visible (Fig. 4b1-3; 4c1-3). In the newly formed cells, the cytoplasm and necks are not yet completely filled with adhesive vesicles. Therefore, the number of PNA positive cells could not be quantified until 72 h. Macif1 immunoreactivity was restricted to the cells in the ventral epidermis. PNA positive cells were found individually within the blastema, with some connections to Macif1 positive cells. In contrast, no anchor cells without a connecting adhesive gland cell were identified. After 72 h of regeneration, the anchor cells formed their characteristic arc. We found that 26 ± 6 (*n* = 10) adhesive gland cells were differentiated. The necks of the adhesive gland cells elongated to their final length of approximately 35 to 50 μm (Fig. 4d1-3). Five days post amputation 45 ± 8 (*n* = 10) adhesive gland cells were regenerated, after 8 days, 85 ± 7 (*n* = 10) were quantified. After 10 days, the tail regeneration was completed and the full number of 127 ± 22 (*n* = 10) was reached.

**Fig. 5:**
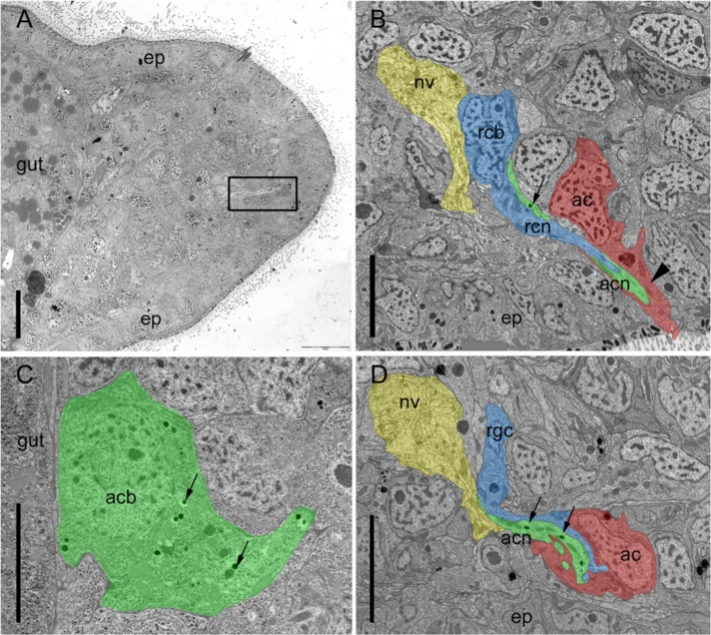
Ultrastructure of *Macrostomum lignano* regenerating adhesive organs after 48 h of tail plate regeneration. Anterior is to the left and dorsal to the top. **a** Overview of the tail plate 48 h post amputation. Rectangle indicates a regenerating adhesive organ. **b** Differentiated adhesive organ with anchor cell emerging through the epidermis. Arrowhead indicates intermediate filaments in the anchor cell. Arrow indicates a vesicle of the adhesive gland cell. **c** Adhesive gland cell body positioned at the basis of the blastema, next to the gut, with adhesive vesicles (arrows). **d** Immature adhesive organ, with anchor cell located beneath the epidermal layer. Note that the anchor cell is still lacking intermediate filaments and microvilli. Arrows indicate vesicles of the adhesive gland cell. *Ac* anchor cell, *acb* adhesive gland cell body, *acn* adhesive gland cell neck, *ep* epidermis, *nv* nerve, *rcb* releasing gland cell body, *rcn* releasing gland cell neck. Scale bars: (**a**) 10 μm, (**b**-**d**) 5 μm

For a detailed morphological analysis and to localize the releasing gland cells, TEM serial sagittal sections of a specimen after 48 h of tail regeneration were made (Fig. 5). We identified one completely differentiated and four immature adhesive organs. The completely differentiated adhesive organ shared all characteristic features described earlier [11]. The anchor cell body was located in the ventral epidermis, with the nucleus situated below the body wall musculature (Fig. 5b). Its cytoplasm was filled with intermediate filaments, and the microvilli collar protruded from the epidermis. Both gland cell necks penetrated the anchor cell and contained vesicles. The releasing gland cell body was located next to the nerve loop and in close proximity to the anchor cell (Fig. 5b). This was found in all identified releasing gland cell bodies (three cells from different adhesive organs). The cell bodies of the adhesive gland cells (Fig. 5c) were located more anteriorly in the blastema compared to the releasing gland cells and anchor cells. The adhesive gland cell necks that extended through the blastema connected with one releasing gland cell neck, and together they penetrated one anchor cell (Fig. 5b, d). In immature adhesive organs (4 out of 4), the anchor cell was already penetrated by the two gland cells while still located below the epidermis (Fig. 5d). The immature anchor cells did not emerge at the epidermis surface, had no intermediate filaments, and their microvilli collar was still missing. In contrast, both gland cells were already differentiated and produced their characteristic vesicles (Fig. 5d). In summary, these findings indicate that the two gland cells differentiate earlier than the anchor cell and penetrate the latter before it migrates into the epidermis and fully differentiates.

### Can adhesive gland cell necks regenerate and penetrate a novel anchor cell?

**Fig. 6:**
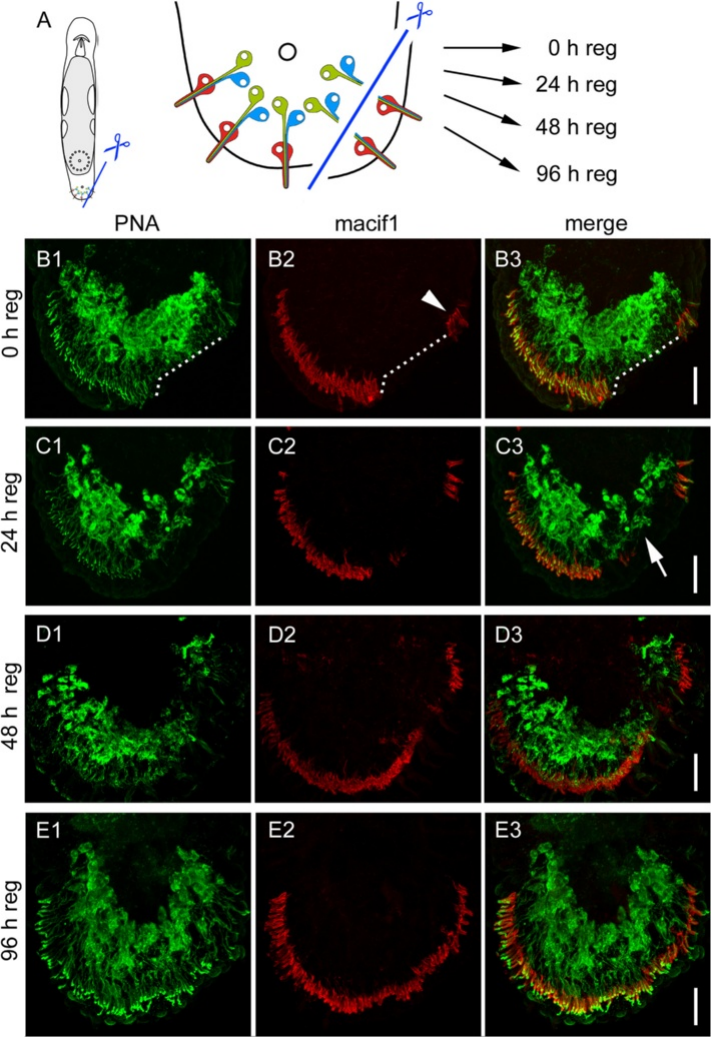
Regeneration of adhesive organs after partial amputation. **a** Schematic drawing of *Macrostomum lignano* with indication of the amputation level. The specimen were amputated on one side of the tail plate, removing the epidermal layer and the anchor cells. The cut went through the adhesive gland cell necks, but left the adhesive gland cell bodies intact. **b**-**e** PNA and Macif1 double staining of regenerating specimen at (B) 0 h, (C) 24 h, (D) 48 h and (E) 96 h after partial amputation. **b** The dotted line indicates the area of the cut. Arrowhead indicates anchor cells that were not affected by the cut. **c** After 24 h of regeneration the staining of adhesive gland cell vesicles (PNA) was reduced at the amputation side (arrow). **d** After 48 h the first anchor cells and adhesive gland cell necks were rebuilt. **e** After 96 h the regeneration of the adhesive organs was completed. Scale bars: 20 μm

**Fig. 7:**
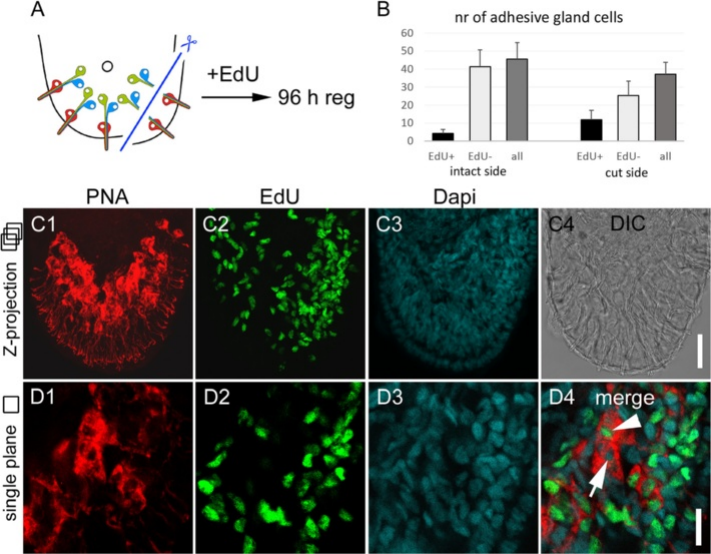
Continuous EdU treatment after partial amputation. **a** Schematic drawing of the tail plate with indication of the amputation level. **b** Number of EdU-positive and -negative adhesive gland cells after 96 h of regeneration on the uncut side and cut side. The numbers are based on the average of 12 specimen, error bars indicate the standard deviation. **c**-**d** Representative images of PNA, EdU, and Dapi staining after 96 h of regeneration. Note the higher number of EdU positive cells on the side of amputation. Images in (**c**) are maximal intensity projections of optical sections and in (**d**) one single optical section is shown. The arrowhead indicates an EdU-positive adhesive gland cell, the arrow highlights an EdU-negative adhesive gland cell. Scale bars: (C) 20 μm, (D) 10 μm

**Fig. 8:**
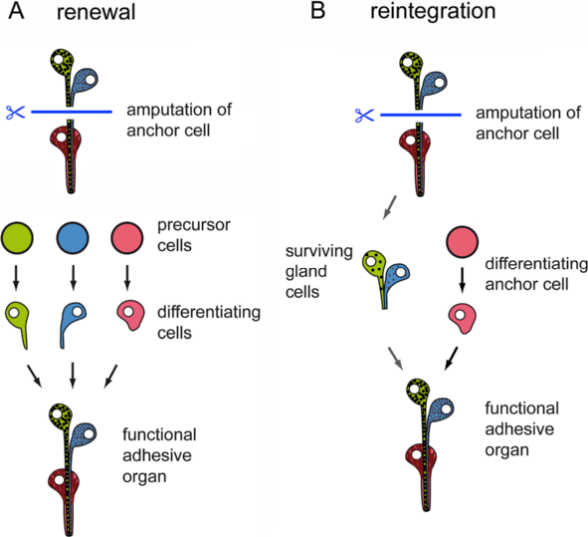
Proposed models for adhesive organ regeneration after anchor cell amputation. **a** Renewal of all three cells. **b** Survival of gland cells and reintegration of a newly formed anchor cell

After tail amputation, all three cell types of adhesive organs are lost and need to be rebuilt. However, what happens if only the anchor cells are amputated? Can the gland cells rebuild an adhesive organ through the inclusion of a stem cell that differentiates into an anchor cell, or do the gland cells degenerate and is a completely new adhesive organ built? To test these possibilities, we performed a partial amputation of the tail plate, resulting in the loss of anchor cells on the amputation side (Fig. 6a). The amputation removed the anchor cells on one half of the tail plate and also cut the corresponding necks of the adhesive gland cells. However, the gland cell bodies remained intact (Fig. 6b). Due to the half-moon shaped orientation of the adhesive organs, the straight cut did not impact all the adhesive organs, but left some of the most anteriorly located adhesive organs unaffected (Fig. 6b2). After 24 h of regeneration, the amount of PNA labelled cells at the amputation site was reduced (Fig. 6c1-3). The uncut side of the tail plate showed no difference with specimen in homeostasis (Fig. 6c1-3). Forty-eight hours post amputation, the first anchor cells were rebuilt and connected with the adhesive gland cell necks (Fig. 6d1-3). After 96 h, the adhesive organs were regenerated, and the amputated side was almost indistinguishable (Fig. 6e). To identify the newly formed adhesive gland cells, animals in homeostasis and partially amputated worms were continuously treated with 5-ethynyl-2′-deoxyuridine (EdU) for 96 h and counterstained with PNA and DAPI (Fig. 7). EdU is an analogue to thymidine, and it is incorporated into the DNA during DNA synthesis (for EdU staining in *M. lignano*, see e.g. [9]). We quantified EdU-positive and -negative adhesive gland cells in the tail plate of homeostasis animals. During homeostasis, on average, 10.6 % (10.6 ± 4.6 cells out of 110.4 ± 13.2, *n* = 10) of the adhesive gland cell bodies were EdU positive after 96 h of EdU treatment, indicating a continuous renewal of adhesive gland cells. In the partially amputated animals, we distinguished between the cut and uncut halves of the tail plate using the male opening as a reference for the midline of the animals (Fig. 7a). A similar number of 9.4 % (4.3 ± 2.1 cells out of 45.6 ± 9.0, *n* = 12) of labelled adhesive gland cells were EdU positive at the intact side of the partially amputated animals. In contrast, on the amputated side, 31.8 % (11.8 ± 5.3 cells out of 37.2 ± 6.7, *n* = 12) of the adhesive gland cells showed an EdU-positive nucleus. Although the number of EdU-positive adhesive gland cells at the amputated side was increased, the majority of the adhesive gland cells (68.2 %) had an EdU-negative nucleus. We confirmed that after whole tail plate amputation, 100 % of the labelled adhesive gland cells (155 cells, *n* = 6) were EdU positive after 96 h of regeneration (Additional file 10: Figure S9). These findings indicate that some adhesive gland cells were renewed after amputation, but others were able to survive, elongate their gland necks, and penetrate a newly formed anchor cell. In summary, these results demonstrate that adhesive gland cell regeneration can proceed in two ways (Fig. 8). First, all three cells are newly built and form an adhesive organ *de novo* (Fig. 8a). Second, if only the anchor cells are amputated, the gland cells can survive and regrow their necks to penetrate a newly built anchor cell (Fig. 8b).

To clarify whether the regrowth of other gland necks is a common ability in *M. lignano*, we amputated animals anteriorly to the brain and visualized the regeneration of the gland cell necks using the lectin sWGA (Additional file 11: Figure S10). After amputation, the gland cell bodies remained, and their necks elongated with the regenerating rostrum (Additional file 11: Figure S10). Therefore, we conclude that outgrowth of gland cell necks can be a general feature of the regenerating tissues of *M. lignano*.

## Discussion

### Lectins as new markers in *Macrostomum lignano*

*Macrostomum lignano* is an emerging model system for studies of stem cell dynamics and regeneration [5, 7–9, 20–28]. Recently, the genome and transcriptomes of *M. lignano* were published, facilitating molecular and genetic studies [29, 30]. However, the visualization of cell types is limited to *in situ* hybridization [24] and a few specific antibodies [7, 13, 14]. With this study, we add commercially available lectins as simple-to-use and inexpensive labelling reagents to the methodical toolbox of *M. lignano*. Five lectins label various types of secretory gland cells throughout the animal. Additionally, lectin labelling revealed four new types of frontal glands in the anterior part of the animal. The function of these gland cells and the composition of their secretions is currently unknown. In *Schmidtea mediterranea,* the proteinaceous component of secreted mucus was identified [31], and the involvement of the secretions in locomotion, innate immunity, adhesion, and protection against environmental reactive oxygen species was predicted. Secreted proteins showed a high similarity between parasitic- and free living flatworms [31] and may also be conserved in *M. lignano*. Lectins used in combination with in situ hybridization could help to map the expression of secreted proteins to a specific gland cell type and thereby reveal their function. Additionally, we showed that lectins can be combined with EdU labelling and therefore enable the visualization of cell turn-over and renewal. These approaches will further enable the investigation of gene function during differentiation and regeneration processes.

### PNA labelling of adhesive gland cells

Many marine organisms rely on adhesive secretions to attach themselves temporary or permanently to the substrate [32]. Most of these adhesives consist of various proteins, either alone or in combination with other components. Glycoproteins have been identified in the adhesives of several marine organisms, such as barnacles [33, 34], the sea star *Asterias rubens* [19, 35], the green mussel *Perna viridis* [36, 37], and the green alga *Ulva* [38, 39]. In the sea star *A. rubens*, 11 lectins labelled the disc epidermis at the level of adhesive cells, and four (DBA, WGA, RCA, and Con A) additionally labelled the secreted adhesive material [19]. In *M. lignano,* PNA and RCA labelled the adhesive gland cells, along with other structures. The labelling of the adhesive gland cells with PNA was restricted to their secretory vesicles, suggesting that the PNA labelled glycoconjugate is secreted and part of the adhesive. The subcellular localization of the RCA labelling could not be visualized. Both PNA and RCA also label the subepidermal marginal adhesive glands in the planarian *S. mediterranea* [17]. Whether the corresponding glycoconjugates are conserved and involved in the adhesive glue is currently unknown. There are eight additional lectins leading to a staining in the adhesive glands of *S. mediterranea*. Four of these (DBA, WGA, sWGA, and SBA) were also tested in *M. lignano* and led to a different staining result.

### Adhesive organs as model for organogenesis

Flatworms are well known for their capacity to regenerate large body parts. The potential to investigate naturally occurring organ regeneration in an *in vivo* system led to several regeneration studies within the last few years (reviewed in [40]). In the past years, the central nervous system was the favourite model system for whole organ regeneration (reviewed in [41]). Yet the broad range of cell types and differently expressed genes within this system made analyses challenging [42]. Recently, there has been a trend in flatworm regeneration research to investigate simpler organ systems, such as the excretory system [42, 43], the optic cups [44–47], the pharynx [48], and the intestine [49, 50]. Most regeneration studies were focused on free-living triclads such as *S. mediterranea* and *Dugesia japonica*, whereas the less derived Macrostomorpha [51–53] gained less attention. One great advantage of studying the regeneration of adhesive organs in a species of *Macrostomum* is its simple structure of only three interacting cells [11]. Few systems are equally simple and accessible but still with the complexity of multi-cellular organs. Even in closely related families of the Macrostomida, the adhesive organs exhibit a higher complexity. For example, in the family of *Microstomum,* the releasing glands form branches; in *Bradynectes,* the microvilli number and arrangement vary highly between single organs; and in *Myozona,* two adhesive glands are associated with one releasing gland and one anchor cell [12, 54, 55]. In higher orders of Platyhelminthes, including Proseriata, Tricladida, and Rhabdocoela, the adhesive organs form a relatively large adhesive field consisting of numerous anchor cells and gland neck openings. The gland necks are highly branched and penetrate several anchor cells. In contrast to *M. lignano*, adhesive papillae are also present on the lateral sides of the body or even encircle it [12, 55].

### Regeneration of adhesive organs

In earlier studies of *M. lignano*, the number of adhesive papillae was used as an indication for complete tail plate regeneration [6, 9, 27]. However, as no suitable markers were available, no further investigations on adhesive organ regeneration or differentiation were performed. With the lectin PNA and the Macif1 antibody, we visualized the location of differentiated adhesive gland cells and anchor cells during regeneration. Thus far, no marker for releasing gland cells could be identified. To remove the complete tail plate, we used the same cutting level as has been used in previous studies [9, 27]. The regeneration of adhesive organs requires the differentiation of stem cells towards adhesive- and releasing gland cells and anchor cells. First, one adhesive- and one releasing gland cell differentiate in a temporally and spatially coordinated manner to form the gland cell pair of one adhesive organ. Second, the outgrowing gland cell necks of the adhesive and the releasing glands, which are in direct contact with each other, penetrate an undifferentiated anchor cell. At this point, the future anchor cell is still located within the regeneration blastema and has not reached the epidermal surface and no intermediate filaments are expressed. Finally, the anchor cell integrates into the epidermis and forms junctional complexes with the neighbouring epidermal cells [11]. Then they establish the intermediate filament network in their cytoplasm, with connection to the tail plate ECM occurring via hemidesmosomes [11]. After 10 days of regeneration, all adhesive organs are formed and conjointly give rise to the horse-shoe shaped adhesive system of the tail plate [11].

After amputation of the anchor cells on one side of the tail plate, the majority (68.2 %) of the adhesive gland cells were EdU negative at the amputated site. In contrast, 96 h after amputation of the whole tail plate, all PNA labelled adhesive gland cells had an EdU-positive nucleus. This indicates that some adhesive gland cell bodies were able to survive the partial amputation of their necks and reconnected with a newly formed anchor cell. In *M. lignano,* a pool of neoblasts is present that does not undergo cell division but is ready to differentiate into the required cell types [25, 26]. In addition, neoblasts arrested in G2-phase were predicted [25, 26]. It is possible that some of the EdU-negative adhesive gland cells differentiated from these neoblasts, which did not undergo a round of cell division and are EdU negative. However, it is unlikely that all EdU-negative gland cells originate from this pool of stem cells since their overall number would be too low. The amputation of the anchor cells led to a decrease in PNA labelling after 24 and 48 h at the area of the cutting. As the PNA labelling was restricted to the adhesive gland vesicles, we speculate that after the amputation of their necks, the adhesive gland cells stopped the production of vesicles until their reconnection to a new anchor cell.

## Conclusion

Lectins have been shown to be highly useful tools as markers for tissues and organs in diverse organisms. We now show that a collection of nine lectins can be used to stain specific cell types in *Macrostomum lignano*. We used PNA-based labelling of adhesive gland cells to study the regeneration of adhesive organs in this species. In combined staining with an anchor cell specific antibody, we explored the spatial and temporal formation of the adhesive system upon amputation. Furthermore, we examined the ability of gland cells to regrow amputated gland cell necks. With respect to the adhesive organs, this required the integration of a newly differentiated anchor cell into the rebuilding adhesive organ, as compared to the complete *de novo* formation of adhesive organs after whole tail amputation. Overall, our data can provide a foundation for understanding cell differentiation, cellular interactions, and organ formation.

## Methods

### Animal culture

*Macrostomum lignano* [10] cultures of the inbred line DV1 [56] were kept in petri dishes with nutrient enriched artificial seawater (Guillard’s f/2 medium) [57] and were fed *ad libitum* with the diatom *Nitzschia curvilineata.* Animals were maintained in a climate chamber with 20 °C, 60 % humidity and a 14:10 day-night cycle.

### Lectin histochemistry

Animals were relaxed with 7.14 % MgCl_2_ hexahydrate and then fixed in 4 % formaldehyde (made from paraformaldehyde) in PBS (PFA) for 1 h. Afterwards the specimen were washed six times 10 min in Tris-buffered saline (pH 8.0) supplemented with 5 mM CaCl_2_ and 0.1 % Triton (TBS-T). Buffers were additionally supplemented with 5 mM MnCl_2_ for Con A, LCA, PSA, and PHA-L, and 5 mM MgCl_2_ for GSL I. Unspecific background staining was blocked by pre-incubation in TBS-T containing 3 % *(w/v)* bovine serum albumin (BSA-T) overnight at 4 °C. Biotinylated lectins were diluted in BSA-T to a final concentration of 25 μg/ml and applied to the specimen for 2 h at room temperature. After six washes of 10 min each in TBS-T, the specimen were either incubated for 2 h in Texas-Red-conjugated streptavidin (Vector Laboratories) diluted 1:100 in BSA-T or for 1 h in Dylight488-conjugated-streptavidin (Vector Laboratories) diluted 1:300 in BSA-T at room temperature. After several washing steps in TBS-T, the specimen were mounted in Vectashield and analysed using a Zeiss Axioscope A1 microscope or a Leica SP5 II confocal scanning microscope. Control reactions for PNA labelling were performed by pre-incubating the lectin with its inhibitory monosaccharide D-galactose (0.2 M) for 2 h at 4 °C. For super resolution microscopy, the labelled specimen were mounted in Mowiol and examined with a Leica SP8 gSTED microscope system.

### Double labelling of adhesive gland cells and anchor cells

Anti-Macif1 antibodies were raised in rabbits against the peptide CERSRDQKEIKRLRDE (aa 212 - 226) by Eurogentec. For regeneration experiments, the animals were relaxed with 7.14 % MgCl_2_ hexahydrate, cut at the desired level using a razor blade and immediately transferred to f/2-medium. After different times of regeneration, animals were relaxed with 7.14 % MgCl_2_ hexahydrate and then fixed in 4 % PFA for 1 h. After six washing steps of 10 min each with TBS-T, unspecific background staining was blocked by pre-incubation in 3 % BSA-T for 30 min at room temperature. The specimen were incubated with 1:300 diluted biotinylated lectin PNA (Vector Laboratories) in 3 % BSA-T for 2 h at room temperature. After three 10 min washes in TBS-T, the specimen were incubated for 1 h in Dylight488-conjugated-streptavidin (Vector Laboratories) diluted 1:300 in BSA-T at room temperature. The specimen were washed several times with TBS-T and re-fixed with 4 % PFA for 20 min at room temperature. After several washes with TBS-T, specimen were heated overnight in a 1:10 diluted epitope retrieval solution (DakoCytomation K5336) at 80 °C. After several washing steps with TBS-T, the specimen were blocked in 3 % BSA-T for 4 h at 4 °C. The specimen were then incubated with 1:1000 diluted polyclonal Rabbit-α-macif1 antibody in 3 % BSA-T overnight at 4 °C. After six washes of 10 min each with TBS-T, the specimen were incubated for 1 h in a goat-α-rabbit-TRITC antibody diluted 1:600 in BSA-T at room temperature. After several washing steps with TBS-T, the specimen were mounted in Vectashield and analysed using a Leica SP5 II confocal scanning microscope. Stacks were acquired sequentially and z-projected.

### Double labelling of EdU and adhesive gland cells

Amputated and uncut animals were soaked in the thymidine analogue 5-ethynyl-2′-deoxyuridine (EdU; Invitrogen) at a concentration of 100 μM in f/2 medium for 4 days continuously. Afterwards, the animals were washed several times with f/2 medium, relaxed with 7.14 % MgCl_2_ hexahydrate and fixed in 4 % PFA for 30 min. Lectin labelling was performed as described in the section lectin histochemistry, using Texas-Red-conjugated streptavidin. After lectin labelling, the specimen were washed several times with PBS-T and blocked with Blocking reagent solution I (Applichem) overnight at 4 °C. After several washes in PBS-T, the specimen were incubated in Click-iT® EdU reaction cocktail (concentrations according to manufacturer’s instructions – Invitrogen). DNA was visualized with an addition of DAPI (1 μg/ml in PBS-T) for 30 min at room temperature. After several washes with PBS-T, the specimen were mounted in Vectashield and analysed using a Leica SP5 II confocal scanning microscope. Stacks were acquired sequentially and adhesive gland cells with an EdU-positive and -negative nucleus were counted using ImageJ software.

### Electron microscopy

Chemical fixation of *M. lignano* for transmission electron microscopy was performed as described in previous studies [58]. Animals were relaxed with 7.14 % MgCl_2_ hexahydrate and fixed according to [59]. Specimen were dehydrated in an acetone series, embedded in Polybed 812, cut and double stained with uranyl acetate and lead citrate, and examined with a Zeiss Libra 120 TEM (Zeiss, Germany). For preservation of the glycocalyx (Additional file 8: Figure S7) specimen were fixed with 2.5 % glutaraldehyde in 0.1 M cacodylate buffer for 1 h. After washing with cacodylate buffer, specimen were post-fixed with reduced osmium tetroxide (2 % osmium tetroxide + 3 % potassium ferrocyanide in 0.1 M cacodylate buffer). After washing, specimen were treated with 1 % thiocarbohydrazide at 60 °C. After washing with distilled water, specimen were fixed with 2 % osmium tetroxide. After washing, specimen were en-block stained with 1 % uranylacetate overnight and incubated in lead aspartate for 30 min at 60 °C. After dehydration, specimen were embedded in durcupan epoxy resin. Images were made using the Olympus SiS iTEM 5.0 software and a TRS 2048 high speed camera.

## Abbreviations

EdU, 5-ethynyl-2′-deoxyuridine; gSTED, gated stimulated emission depletion; PNA, *Arachis hypogaea* peanut agglutinin; TEM, Transmission Electron Microscopy.

## Additional files

Additional file 1: Table S1. Lectin binding specificities (modified after [19]). With permission of Springer. (DOC 62 kb) Additional file 2: Figure S1. Schematic drawings of frontal gland types. (A) Overview of all found gland types in the anterior region of the animals. (B-E) Single illustrations of the different frontal glands from type one to four. See text for details. (TIF 5623 kb) Additional file 3: Figure S2. SBA labelling of *Macrostomum lignano*. (A) Overview of a SBA stained adult animal with (A1) a confocal projection, (A2) DIC image, and (A3) overlay. (B1-3) Detail of the posterior end showing the intensive labelled antrum, single stained cement glands, and the weakly stained prostate glands. (C1-3) Higher magnification of a head, revealing a dotted staining in frontal glands 1 and a ubiquitous staining in the pharyngeal gland cell bodies and frontal glands 2 and 4. *An* antrum, *cg* cement glands, *fg* frontal glands, *pg* prostate glands, *ph* pharyngeal glands, *st* stylet. Scale bars: (A) 100 μm, (B-C) 20 μm. (TIF 5721 kb) Additional file 4: Figure S3. GSL I labelling of *Macrostomum lignano*. (A) Overview of a GSL I stained adult animal with (A1) a confocal projection, (A2) DIC image, and (A3) overlay. (B1-3) Detail the most anterior part of the rostrum. The openings of frontal glands 1 emerge from the epidermis on the ventral side of the rostrum, whereas the frontal glands 2 emerge at the margin. (C1-3) Higher magnification of a head, with stained frontal glands 1, 2 and 4. *An* antrum, *rb* rhabdites, *fg* frontal glands, *mo* mouth, *ph* pharyngeal glands. Scale bars: (A) 100 μm, (B-C) 20 μm. (TIF 5127 kb) Additional file 5: Figure S4. sWGA labelling of *Macrostomum lignano*. (A) Overview of a sWGA stained adult animal with (A1) a confocal projection, (A2) DIC image, and (A3) overlay. (B1-3) Detail of frontal gland 3 cell bodies. Note that the sWGA positive cell bodies are located in close proximity to rhammite gland cell bodies. (C1-3) Higher magnification of a head, with stained pharyngeal gland and frontal glands 3. The cell necks of frontal gland cells 3 and rhammite gland cells elongate parallel through the neuropil and the rostrum. *Fg* frontal glands, *ph* pharyngeal glands, *rm* rhammites. Scale bars: (A) 100 μm, (B) 10 μm, (C) 20 μm. (TIF 5832 kb) Additional file 6: Figure S5. RCA labelling of *Macrostomum lignano*. (A) Overview of a RCA stained adult animal with (A1) a confocal projection, (A2) DIC image, and (A3) overlay. Arrowheads indicate the unlabeled epidermis. (B) Detail of a tail plate with intensively labelled prostate glands and adhesive organs. (C1-3) Detail of a developing egg within the antrum. *Ao* adhesive organs, *egg* developing egg, *fgo* female genital opening, *pg* prostate glands, *tp* tailplate. Scale bars: (A) 100 μm, (B-C) 10 μm. (TIF 5160 kb) Additional file 7: Figure S6. LCA and Con A labelling of *Macrostomum lignano*. (A) Overview of a LCA stained adult animal with (A1) a confocal projection, (A2) DIC image, and (A3) overlay. (B1) LCA labelling of the epidermal cell junctions, (B2) Dapi staining of the epidermal nuclei, and (B3) overlay. (C) Overview of a Con A stained adult animal with (C1) a confocal projection, (C2) DIC image, and (C3) overlay. (D1-3) Detail of stained epidermis at the level of the tail plate. Arrow highlights adhesive microvilli. *Nu* nucleus of an epidermal cell. Scale bars: (A, C) 100 μm, (B, D) 10 μm. (TIF 5699 kb) Additional file 8: Figure S7. PHA E labelling of *Macrostomum lignano* and details of the glycocalyx. (A) Overview of a PHA E stained adult animal with (A1) confocal projection, (A2) DIC image, and (A3) overlay. (B1-3) Detail of the stained glycocalyx, with unstained rhabdites penetrating the epidermis. (C) TEM image of adhesive organs at the level of the epidermis. Note the glycocalyx surrounding the microvilli of epidermal cells and adhesive organs. (D) PHA E labelling of adhesive organ microvilli and microvilli of epidermal cells. Arrows indicate the glycocalyx on specialized microvilli of adhesive organs and arrowheads indicate the microvilli of epidermal cells. *Ci* cilia of epidermal cells, *mv* microvilli of epidermal cells, *mvao* microvilli of adhesive organs, *rh* rhabdites. Scale bars: (A) 100 μm, (B-D) 5 μm. (TIF 6869 kb) Additional file 9: Figure S8. PNA labelling of *Macrostomum lignano*. (A) Overview of a PNA stained adult animal with (A1) confocal projection, (A2) DIC image, and (A3) overlay. Arrow indicates weakly stained sperm at the centre of testes. (B1-3) Detail of the stained antrum and surrounding cement glands. (C1-3) Detail of a head with stained pharyngeal glands and frontal glands 1, 2, and 4. (D) Control PNA staining and (E) staining with PNA pre-incubated with its inhibitory monosaccharide D-Galactose. Dotted lines indicate the outline of the animals. *Acg* adhesive gland cells, *an* antrum, *cg* cement glands, *fg* frontal glands, *ph* pharyngeal glands. Scale bars: (A, E) 100 μm, (B, C) 20 μm. (TIF 7252 kb) Additional file 10: Figure S9. Regenerated adhesive gland cells after 4 days of tail plate regeneration during continuous EdU treatment. (A) Confocal projections and a DIC image of a regenerated tail plate. (B) Single plane images of regenerated adhesive gland cells. Note that all adhesive gland cells have an EdU positive nucleus. Scale bars: 20 μm. (TIF 2491 kb) Additional file 11: Figure S10. Regeneration of gland necks after rostrum amputation. Confocal projections and DIC images of regenerating heads after (A) 24, (B) 48, (C) 96, and (D) 240 h of regeneration. Black dashed lines indicate the amputation plane. Scale bars: 20 μm. (TIF 7404 kb)
