# Supplementary material for: Adhesive organ regeneration in Macrostomum lignano
Source: BMC Dev Biol. 2016 Jun 2;16:20. doi: 10.1186/s12861-016-0121-1 (PMC4890501; doi:10.1186/s12861-016-0121-1)
Supplement: Additional file 1: Table S1. — Lectin binding specificities (modified after [19]). With permission of Springer. (DOC 62 kb) [file 12861_2016_121_MOESM1_ESM.doc]

| Lectin name | Acronym | Lectin oligosaccharide specificity (most potent hapten) | Comments |
| --- | --- | --- | --- |
| Concanavaline A | Con A | ; | Interacts with glycoconjugates that have at least 2 nonsubstituted or 2-*O*-substituted α-mannosyl residues. |
| *Lens culinaris* agglutinin | LCA |  | Fuc (α1-6) linked to GlcNac important for a tight binding. |
| *Ulex europaeus* agglutinin I | UEA I | TERFucα(1-2)Gal |  |
| Wheat germ agglutinin | WGA |  | - Binds this structure whether it is substituted by NeuAc residues or not.  - Glycoconjugates (glycoproteins, glycolipids or glycopeptides) with a high density of GlcNAc and NeuAc residues will interact with WGA. |
| Succinylated wheat germ agglutinin | sWGA |  | Same specificity as WGA but does not bind to NeuAc residues. |
| *Sambucus nigra* agglutinin | SNA | TER NeuAc(α2-6)GalNAc | Binds specifically to glycoconjugates containing (α2-6) linked NeuAc but also bing very weakly (α2-3) linked NeuAc. |
| *Maackia amurensis* lectin II | MAL II | TER NeuAcα(2-3)GalNAc | Contrary to SNA, MAL II binds only to (α2-3) linked NeuAc. |
| *Phaseolus vulgaris* erythro agglutinin | PHA-E |  | -GlcNAc linked (β1-4) to the Man (β1-4) and terminal Gal residues required for the binding.  - Binds to bi- and tri-antennary chains. |
| *Phaseolus vulgaris* leuco agglutinin | PHA-L |  | - Terminal Gal residues and Man substituted at positions 2 and 6 are required for the binding  - Binds to tri- and tetra-antennary chains. |
| *Ricinus communis* agglutinin | RCA | TERGalβ(1-4)GlcNAc | Binds also to other glycoconjugates containing (β 1-X) linked Gal residues. |
| *Griffonia* (Bandeiraea) *simplicifolia* lectin I | GSL I | TERGalNAcα (1-3)Galβ(1-3)GlcNAc ; TERGalβ(1-3)Galβ(1-4)GlcNAc | Binds also to other glycoconjugates containing (α1-3) linked GalNAc or (α1-3) linked Gal. |
| *Dolichos biflorus* agglutinin | DBA | TERGalNAcα(1-3)GalNAcα(1-3)Galα (1-4)Galβ(1-4)Glc (Forssman pentasaccharide) | Binds also to other glycoconjugates containing (α1-3) linked GalNAc |
| *Sophora Japonica agglutinin* | SJA | TERGalβ(1-3)GalNAc | Binds also to other glycoconjugates containing (α1-3 or 4) linked GalNAc/GlcNAc. |
| Peanut agglutinin | PNA | Galβ(1-3)GalNAc | - GalNAc alone does not allow the binding.  - Galβ(1-3) is essential for a high affinity binding |
| Soybean agglutinin | SBA | GalNAcα or β-linked ; GalNAcα (1-3)Galβ(1-3)GlcNAc ; GalNAcα (1-3)Gal |  |

Asn: asparagin, Fuc: fucose, Gal:galactose, GalNac: N-acetylgalactosamine, Glc: glucose, GlcNAc: N-acetylglucosamine, Man: mannose, NeuAc: N-Acetylneuraminic acid (or sialic acid), TER: terminal.

**Suppl. Table 1.** Biotinylated lectins employed and their binding specificities (table modified after [11])
