# Supplementary figures and images for: Adhesive organ regeneration in Macrostomum lignano
Source: BMC Dev Biol. 2016 Jun 2;16:20. doi: 10.1186/s12861-016-0121-1 (PMC4890501; doi:10.1186/s12861-016-0121-1)

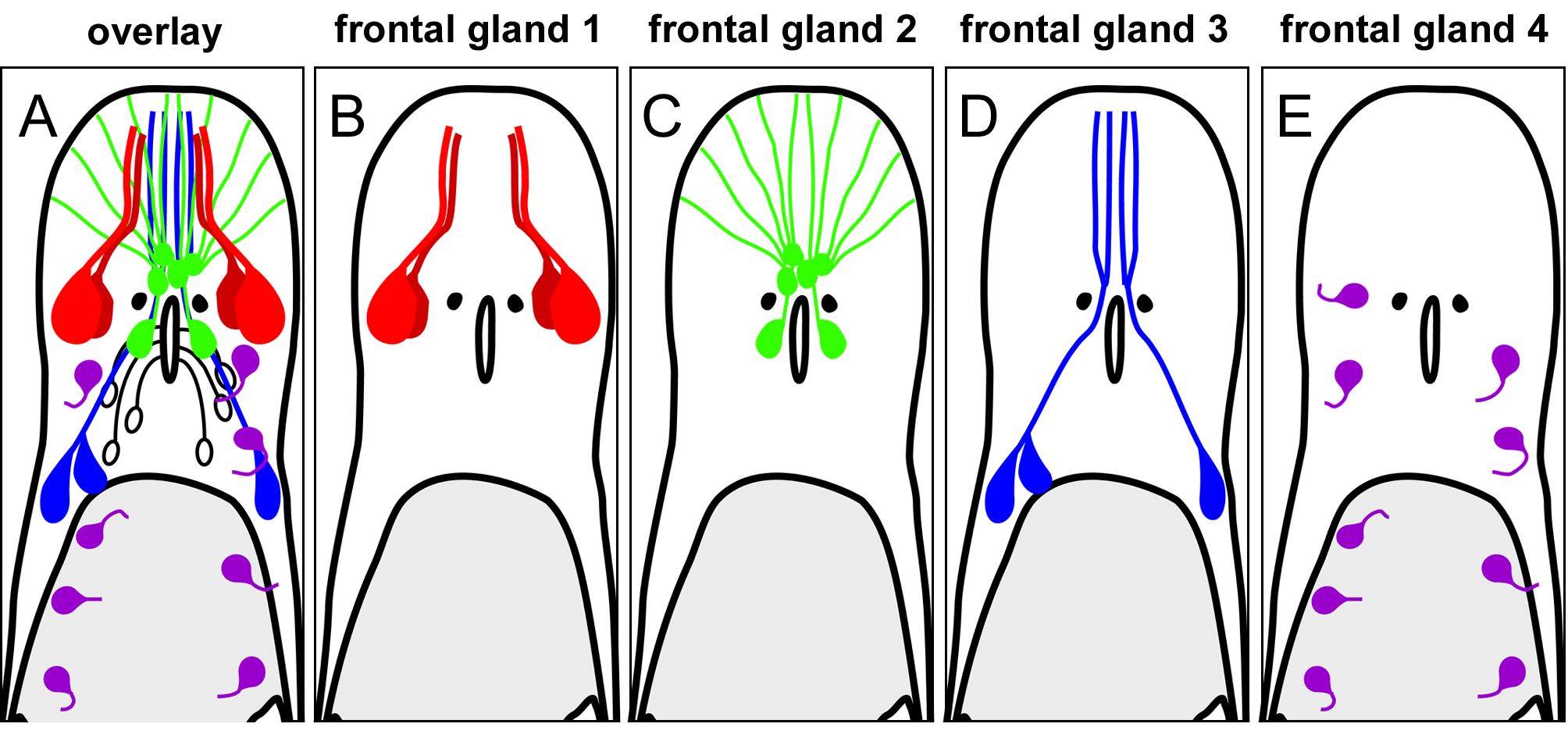

Supplement: Additional file 2: Figure S1. — Schematic drawings of frontal gland types. (A) Overview of all found gland types in the anterior region of the animals. (B-E) Single illustrations of the different frontal glands from type one to four. See text for details. (TIF 5623 kb) [file 12861_2016_121_MOESM2_ESM.tif]

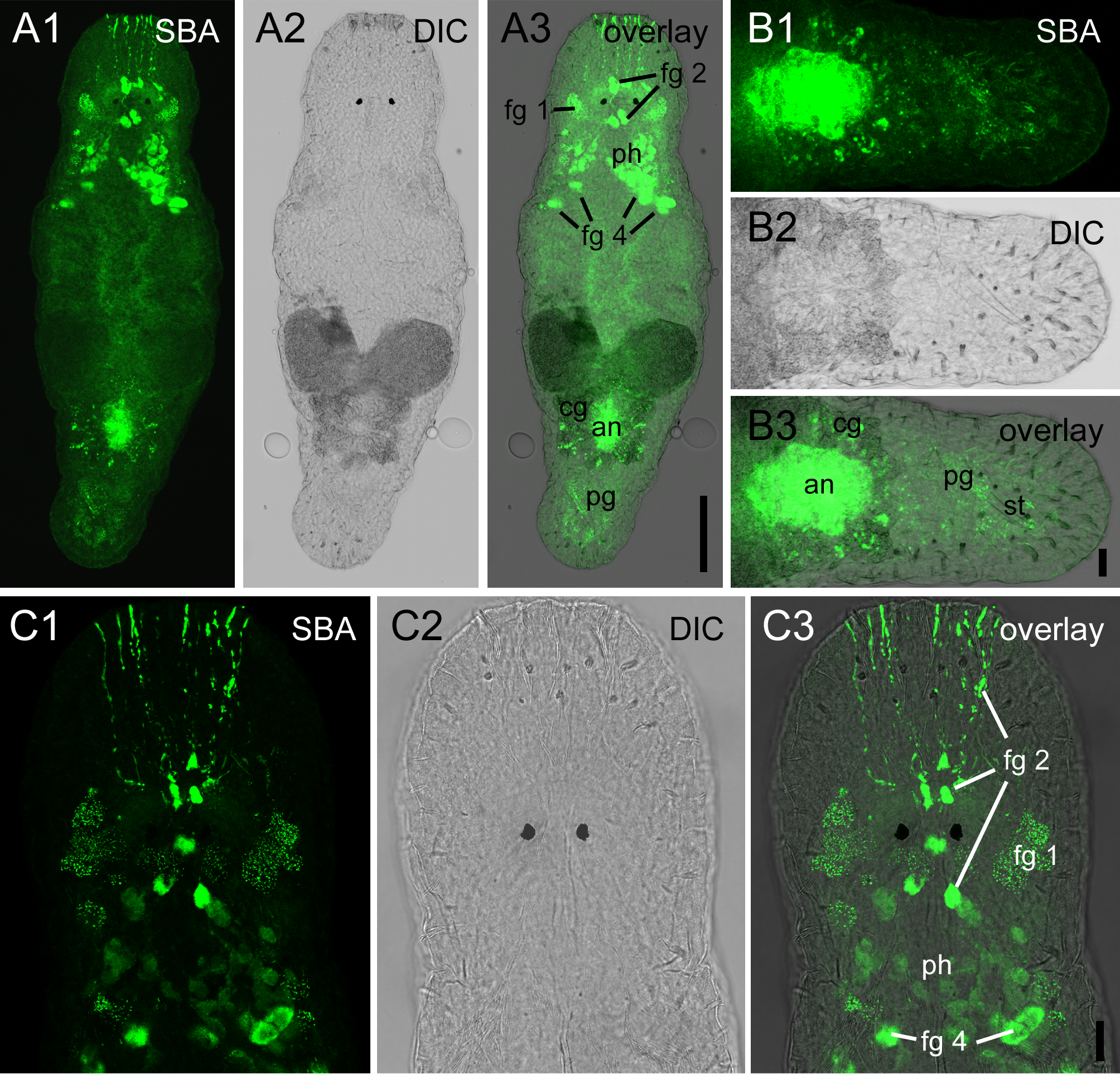

Supplement: Additional file 3: Figure S2. — SBA labelling of Macrostomum lignano. (A) Overview of a SBA stained adult animal with (A1) a confocal projection, (A2) DIC image, and (A3) overlay. (B1-3) Detail of the posterior end showing the intensive labelled antrum, single stained cement glands, and the weakly stained prostate glands. (C1-3) Higher magnification of a head, revealing a dotted staining in frontal glands 1 and a ubiquitous staining in the pharyngeal gland cell bodies and frontal glands 2 and 4. An antrum, cg cement glands, fg frontal glands, pg prostate glands, ph pharyngeal glands, st stylet. Scale bars: (A) 100 μm, (B-C) 20 μm. (TIF 5721 kb) [file 12861_2016_121_MOESM3_ESM.tif]

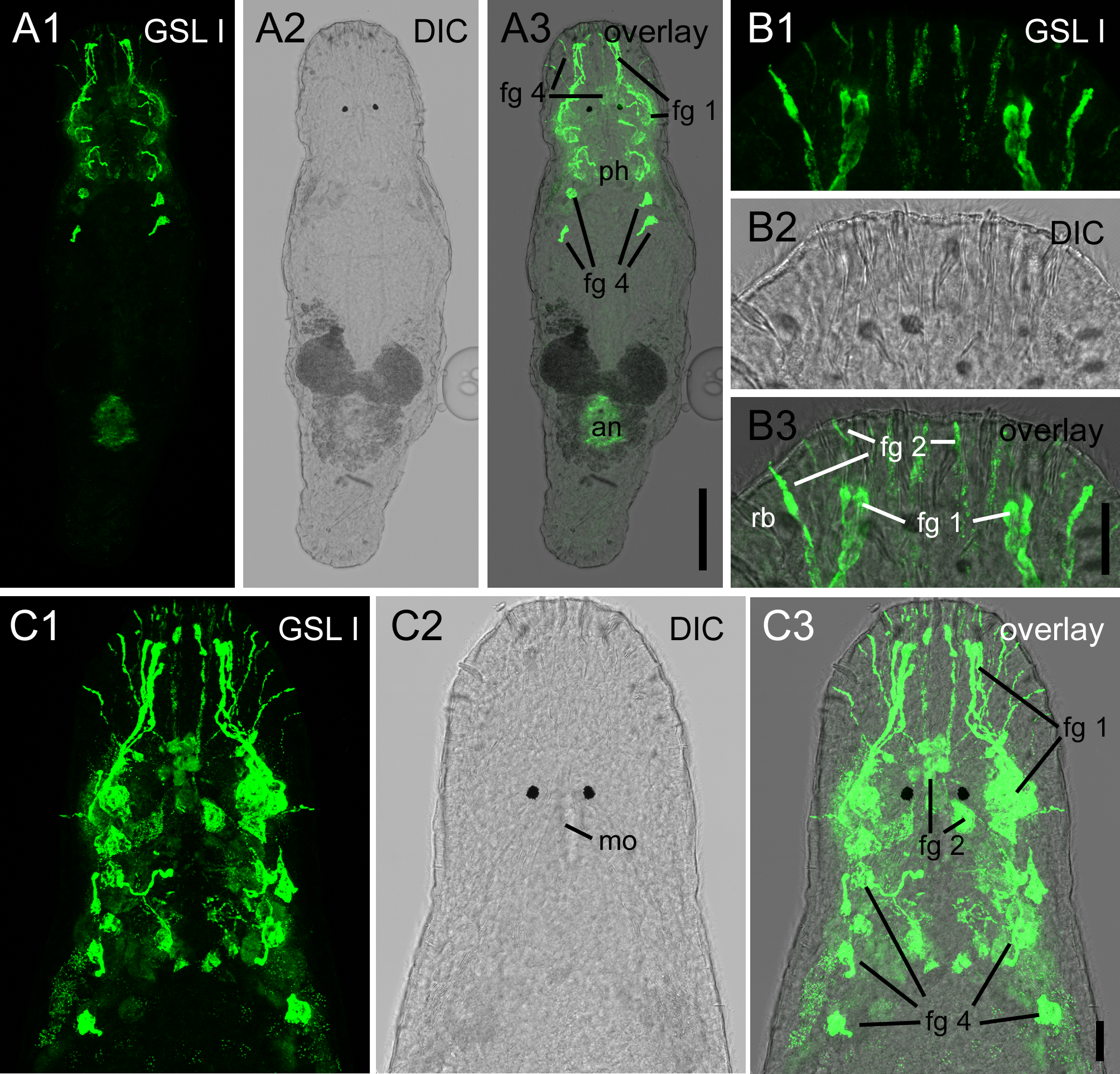

Supplement: Additional file 4: Figure S3. — GSL I labelling of Macrostomum lignano. (A) Overview of a GSL I stained adult animal with (A1) a confocal projection, (A2) DIC image, and (A3) overlay. (B1-3) Detail the most anterior part of the rostrum. The openings of frontal glands 1 emerge from the epidermis on the ventral side of the rostrum, whereas the frontal glands 2 emerge at the margin. (C1-3) Higher magnification of a head, with stained frontal glands 1, 2 and 4. An antrum, rb rhabdites, fg frontal glands, mo mouth, ph pharyngeal glands. Scale bars: (A) 100 μm, (B-C) 20 μm. (TIF 5127 kb) [file 12861_2016_121_MOESM4_ESM.tif]

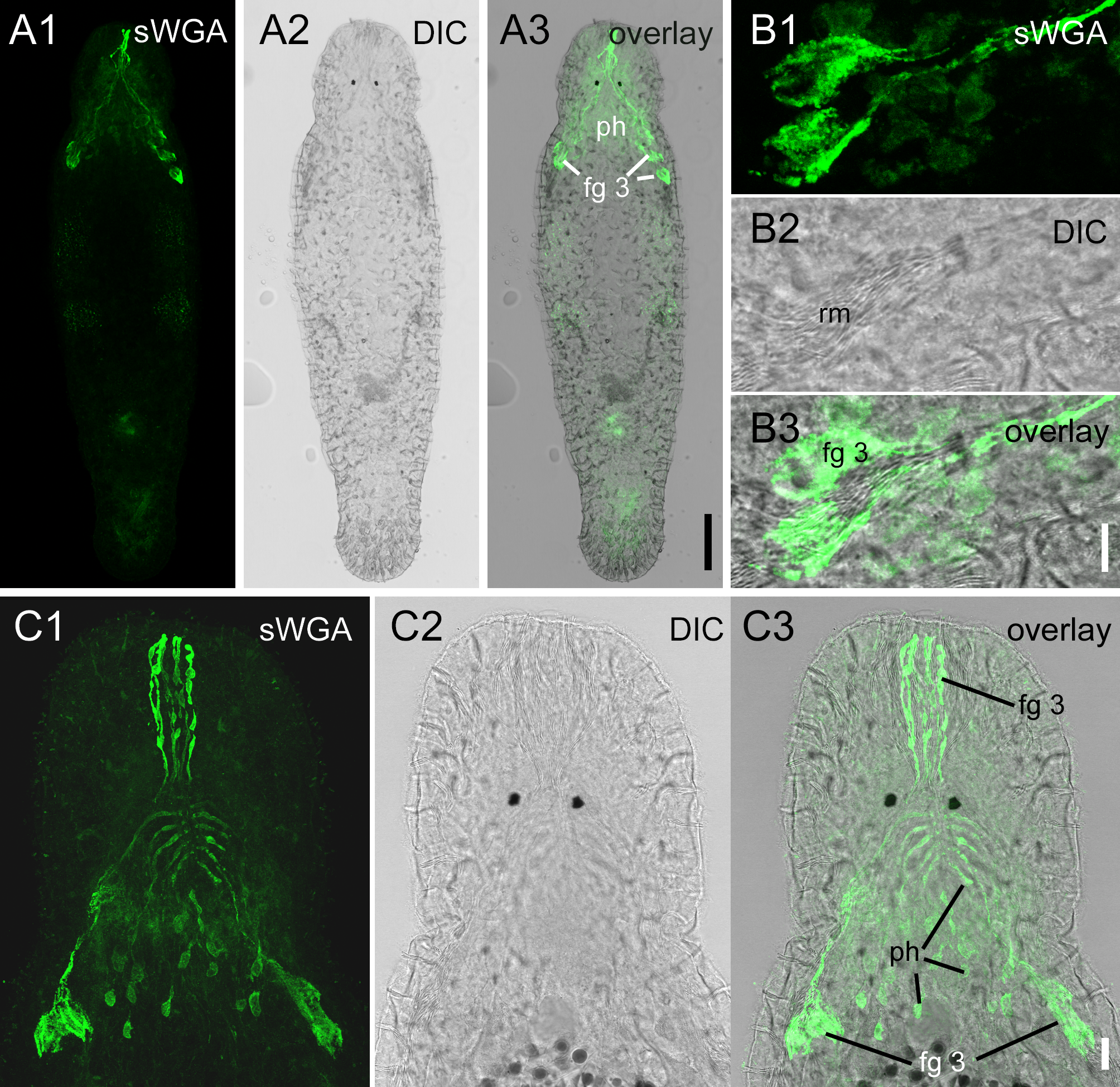

Supplement: Additional file 5: Figure S4. — sWGA labelling of Macrostomum lignano. (A) Overview of a sWGA stained adult animal with (A1) a confocal projection, (A2) DIC image, and (A3) overlay. (B1-3) Detail of frontal gland 3 cell bodies. Note that the sWGA positive cell bodies are located in close proximity to rhammite gland cell bodies. (C1-3) Higher magnification of a head, with stained pharyngeal gland and frontal glands 3. The cell necks of frontal gland cells 3 and rhammite gland cells elongate parallel through the neuropil and the rostrum. Fg frontal glands, ph pharyngeal glands, rm rhammites. Scale bars: (A) 100 μm, (B) 10 μm, (C) 20 μm. (TIF 5832 kb) [file 12861_2016_121_MOESM5_ESM.tif]

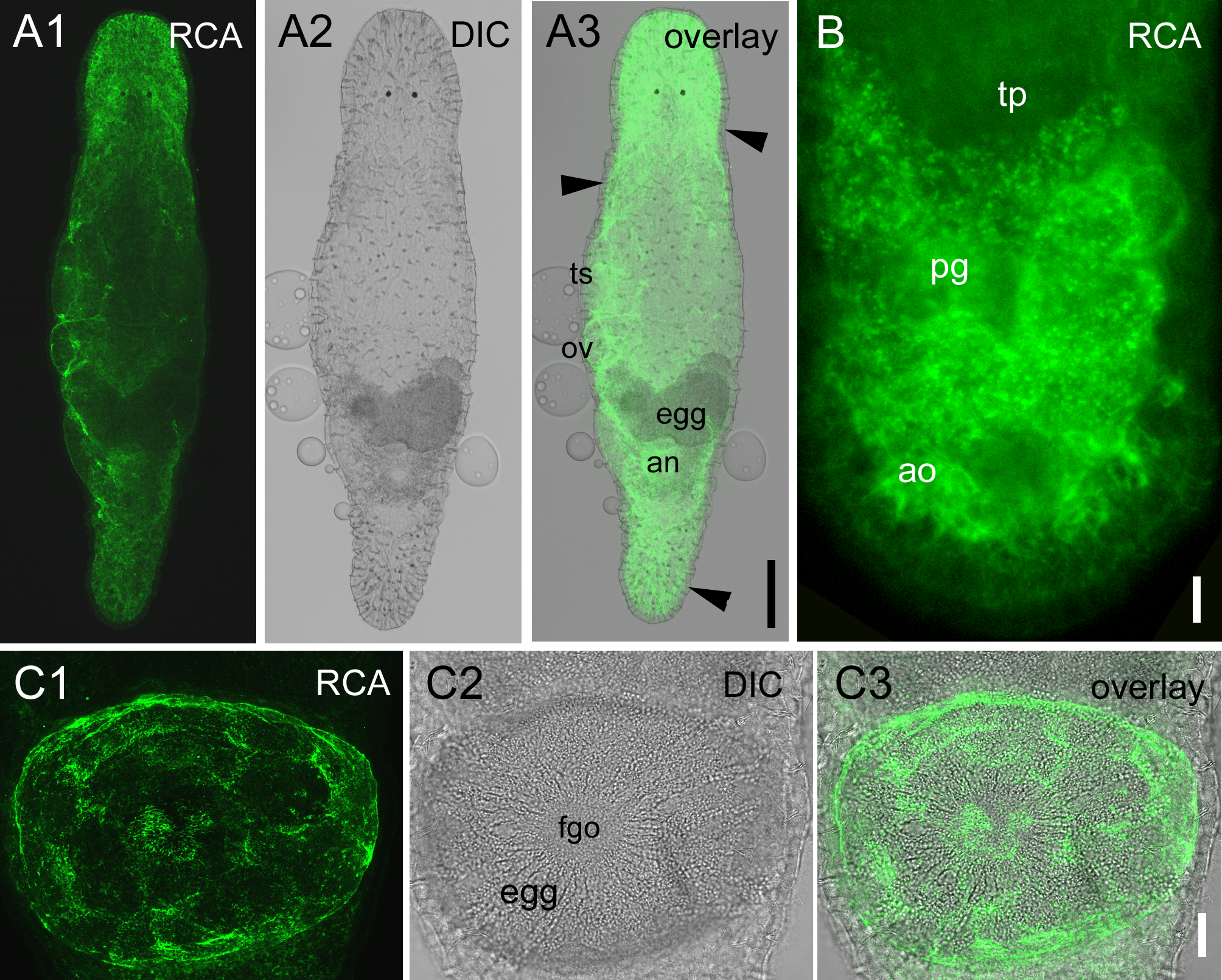

Supplement: Additional file 6: Figure S5. — RCA labelling of Macrostomum lignano. (A) Overview of a RCA stained adult animal with (A1) a confocal projection, (A2) DIC image, and (A3) overlay. Arrowheads indicate the unlabeled epidermis. (B) Detail of a tail plate with intensively labelled prostate glands and adhesive organs. (C1-3) Detail of a developing egg within the antrum. Ao adhesive organs, egg developing egg, fgo female genital opening, pg prostate glands, tp tailplate. Scale bars: (A) 100 μm, (B-C) 10 μm. (TIF 5160 kb) [file 12861_2016_121_MOESM6_ESM.tif]

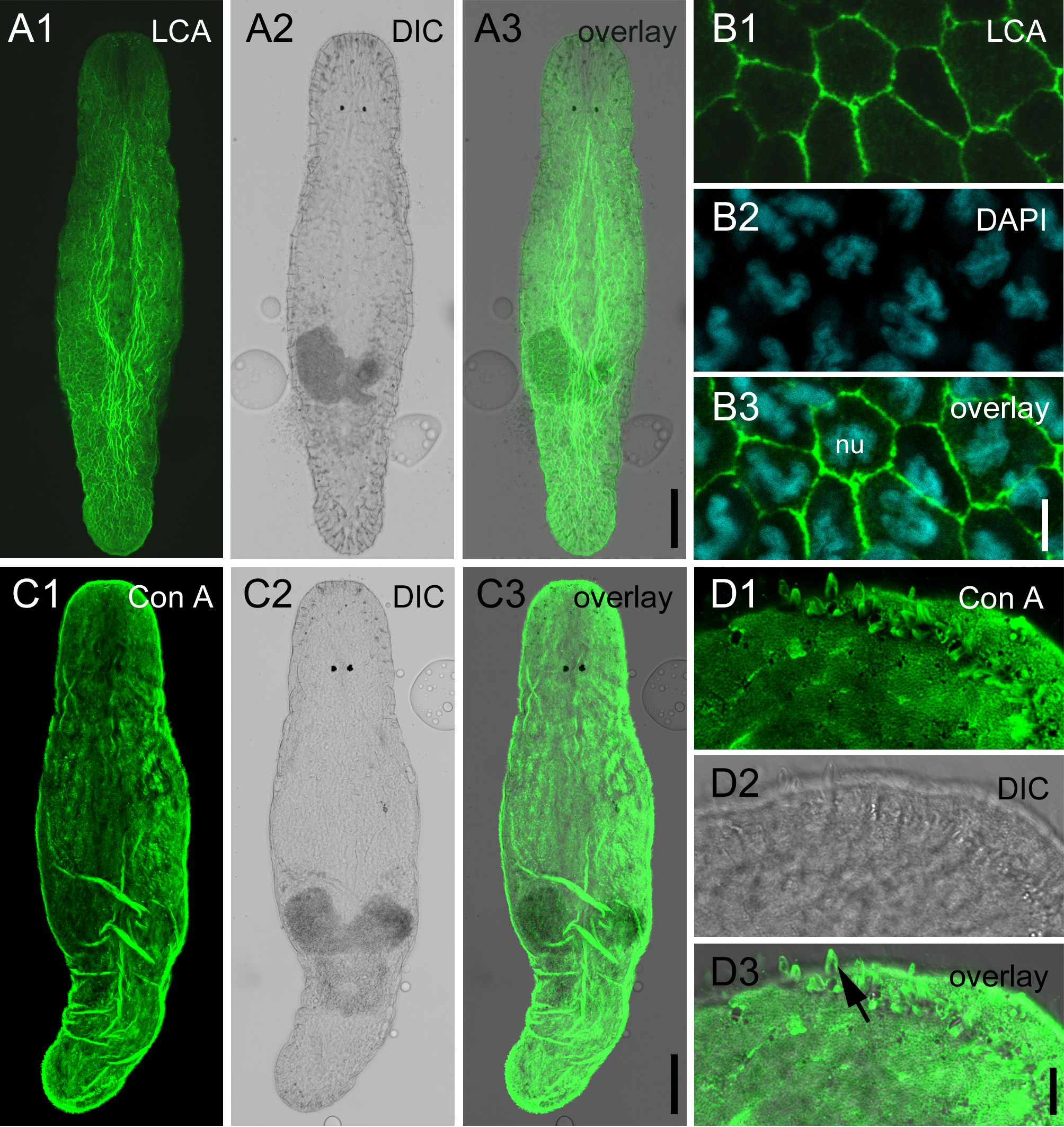

Supplement: Additional file 7: Figure S6. — LCA and Con A labelling of Macrostomum lignano. (A) Overview of a LCA stained adult animal with (A1) a confocal projection, (A2) DIC image, and (A3) overlay. (B1) LCA labelling of the epidermal cell junctions, (B2) Dapi staining of the epidermal nuclei, and (B3) overlay. (C) Overview of a Con A stained adult animal with (C1) a confocal projection, (C2) DIC image, and (C3) overlay. (D1-3) Detail of stained epidermis at the level of the tail plate. Arrow highlights adhesive microvilli. Nu nucleus of an epidermal cell. Scale bars: (A, C) 100 μm, (B, D) 10 μm. (TIF 5699 kb) [file 12861_2016_121_MOESM7_ESM.tif]

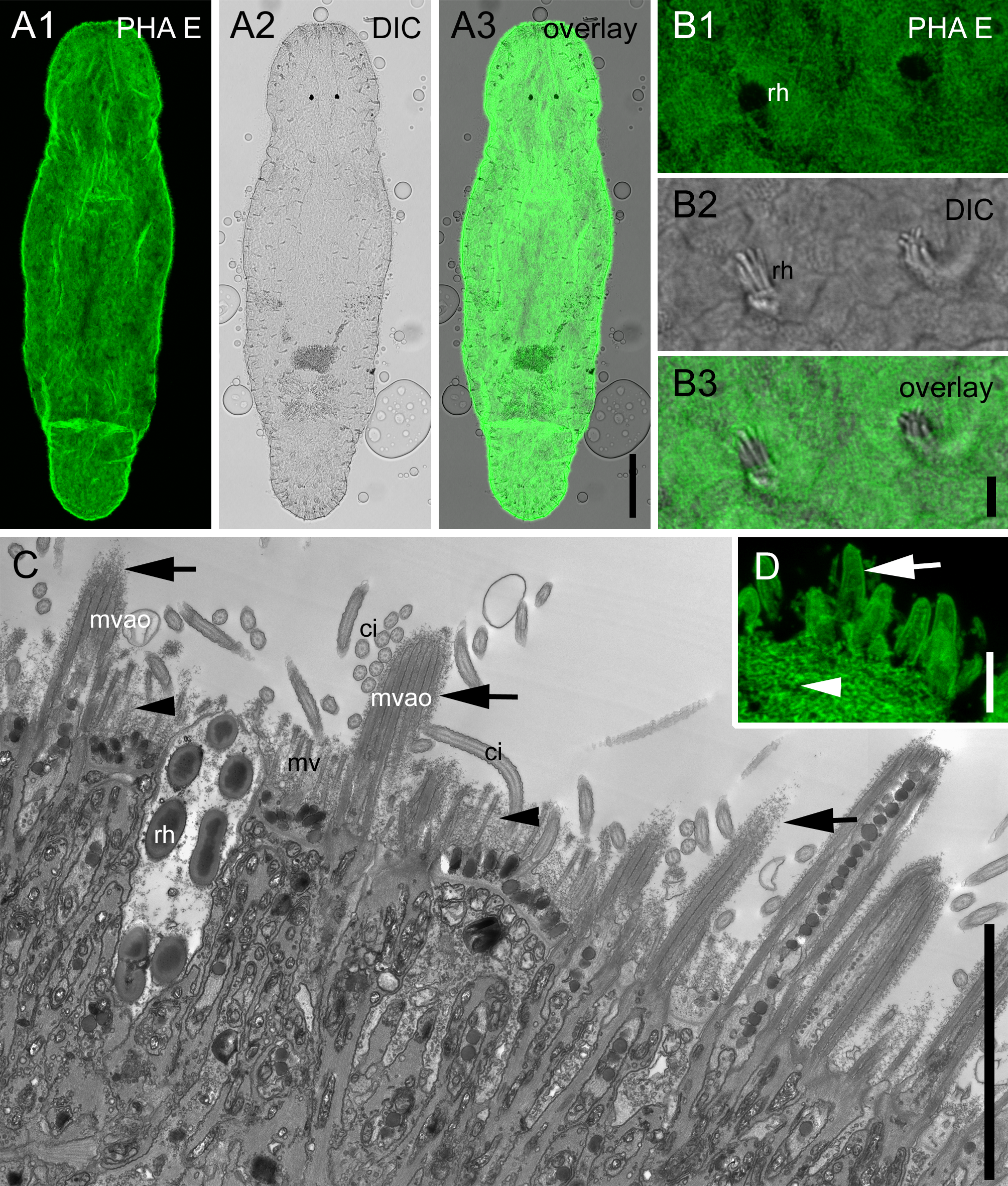

Supplement: Additional file 8: Figure S7. — PHA E labelling of Macrostomum lignano and details of the glycocalyx. (A) Overview of a PHA E stained adult animal with (A1) confocal projection, (A2) DIC image, and (A3) overlay. (B1-3) Detail of the stained glycocalyx, with unstained rhabdites penetrating the epidermis. (C) TEM image of adhesive organs at the level of the epidermis. Note the glycocalyx surrounding the microvilli of epidermal cells and adhesive organs. (D) PHA E labelling of adhesive organ microvilli and microvilli of epidermal cells. Arrows indicate the glycocalyx on specialized microvilli of adhesive organs and arrowheads indicate the microvilli of epidermal cells. Ci cilia of epidermal cells, mv microvilli of epidermal cells, mvao microvilli of adhesive organs, rh rhabdites. Scale bars: (A) 100 μm, (B-D) 5 μm. (TIF 6869 kb) [file 12861_2016_121_MOESM8_ESM.tif]

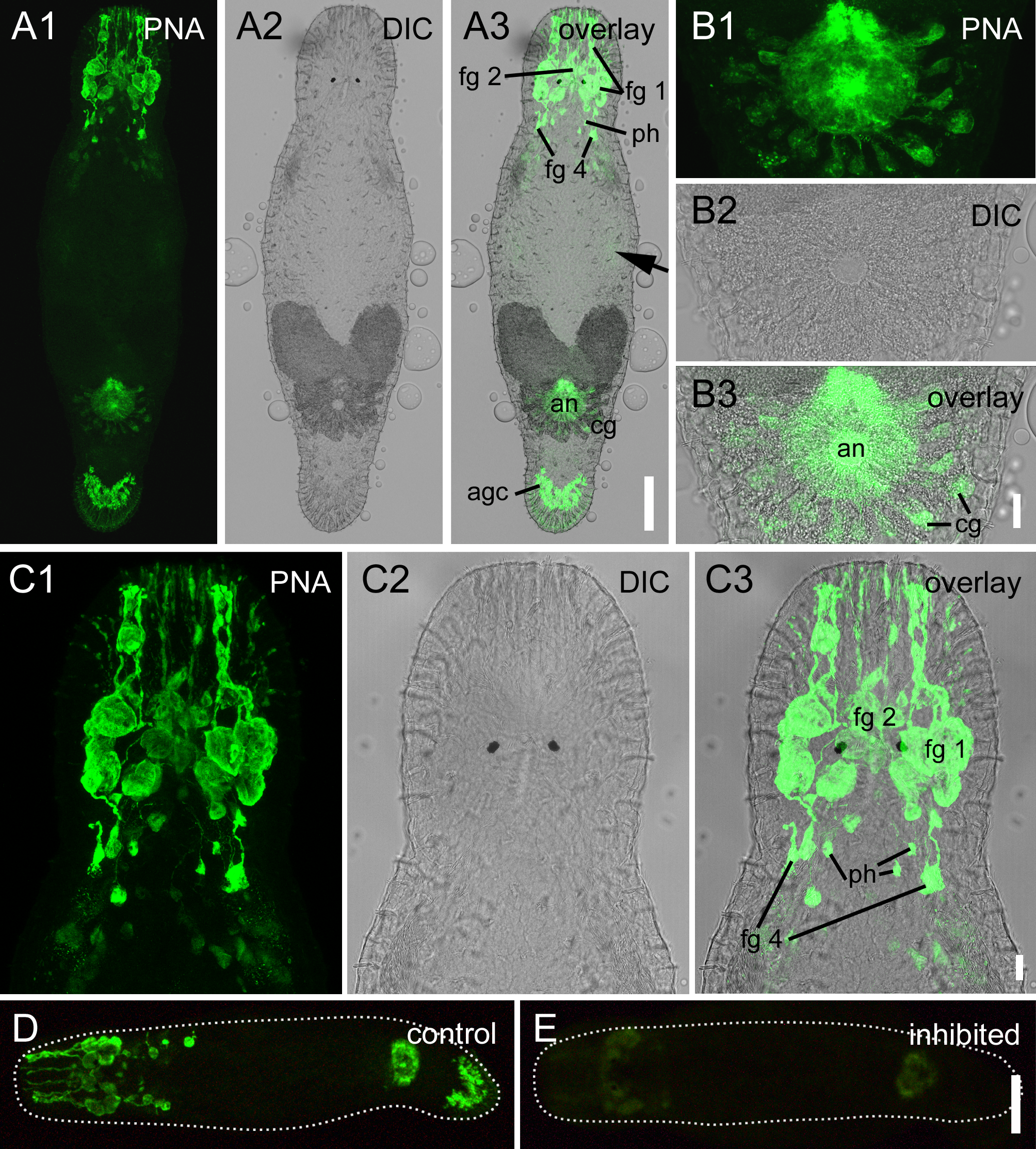

Supplement: Additional file 9: Figure S8. — PNA labelling of Macrostomum lignano. (A) Overview of a PNA stained adult animal with (A1) confocal projection, (A2) DIC image, and (A3) overlay. Arrow indicates weakly stained sperm at the centre of testes. (B1-3) Detail of the stained antrum and surrounding cement glands. (C1-3) Detail of a head with stained pharyngeal glands and frontal glands 1, 2, and 4. (D) Control PNA staining and (E) staining with PNA pre-incubated with its inhibitory monosaccharide D-Galactose. Dotted lines indicate the outline of the animals. Acg adhesive gland cells, an antrum, cg cement glands, fg frontal glands, ph pharyngeal glands. Scale bars: (A, E) 100 μm, (B, C) 20 μm. (TIF 7252 kb) [file 12861_2016_121_MOESM9_ESM.tif]

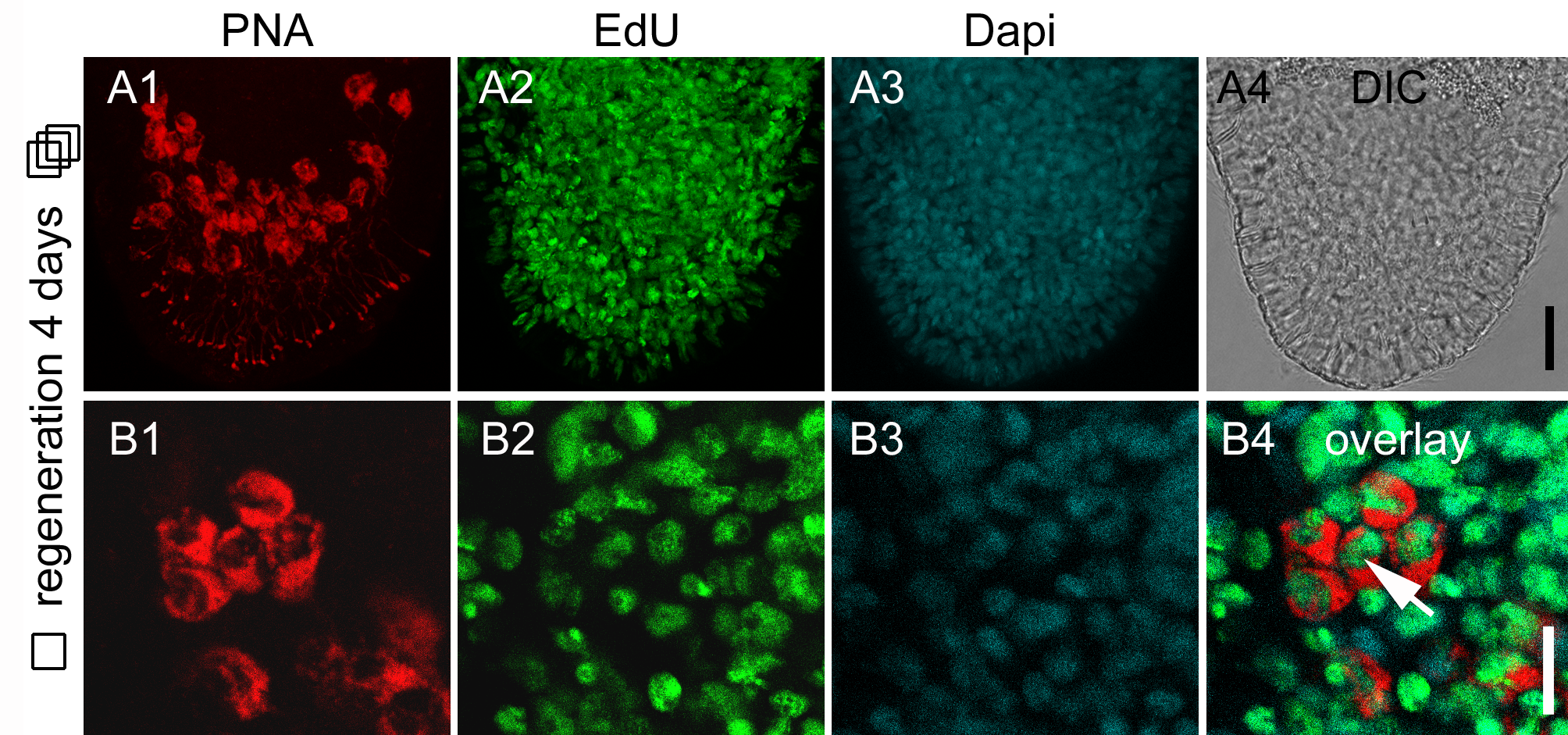

Supplement: Additional file 10: Figure S9. — Regenerated adhesive gland cells after 4 days of tail plate regeneration during continuous EdU treatment. (A) Confocal projections and a DIC image of a regenerated tail plate. (B) Single plane images of regenerated adhesive gland cells. Note that all adhesive gland cells have an EdU positive nucleus. Scale bars: 20 μm. (TIF 2491 kb) [file 12861_2016_121_MOESM10_ESM.tif]

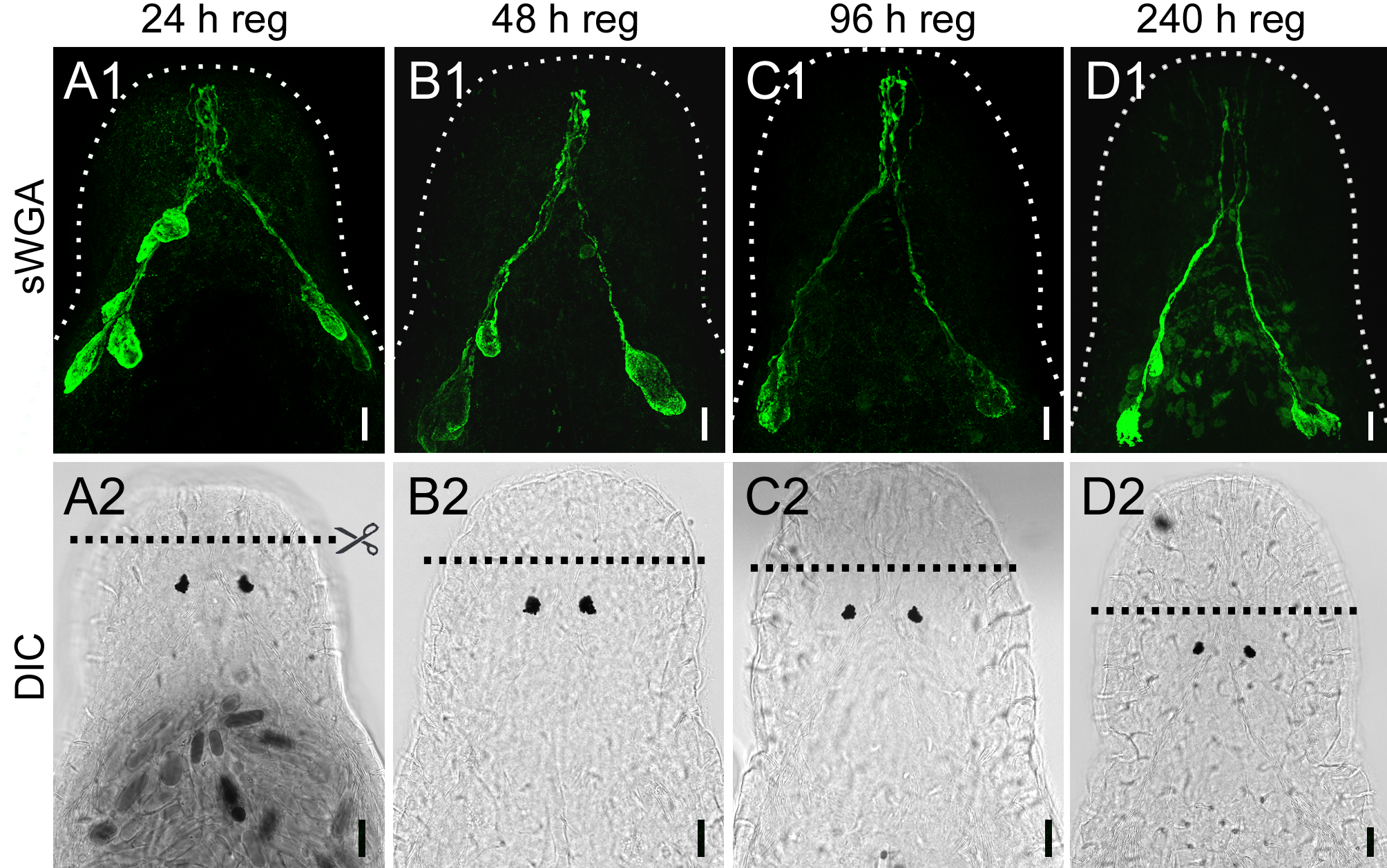

Supplement: Additional file 11: Figure S10. — Regeneration of gland necks after rostrum amputation. Confocal projections and DIC images of regenerating heads after (A) 24, (B) 48, (C) 96, and (D) 240 h of regeneration. Black dashed lines indicate the amputation plane. Scale bars: 20 μm. (TIF 7404 kb) [file 12861_2016_121_MOESM11_ESM.tif]
